# Improving accuracy in genome-wide association studies: a two-step approach for handling below limit of detection biomarker measurements

**DOI:** 10.1093/nargab/lqaf201

**Published:** 2025-12-31

**Authors:** Yaqi A Deng, Torgny Karlsson, Åsa Johansson

**Affiliations:** Department of Immunology, Genetics and Pathology, Science for Life Laboratory, Uppsala University, 75108 Uppsala, Sweden; Department of Immunology, Genetics and Pathology, Science for Life Laboratory, Uppsala University, 75108 Uppsala, Sweden; Department of Immunology, Genetics and Pathology, Science for Life Laboratory, Uppsala University, 75108 Uppsala, Sweden

## Abstract

Advances in high-throughput technologies enable large-scale studies on genomics and molecular phenotypes. However, the trade-off between quality and quantity reduces assay sensitivity, and several measurements in large-scale proteomics and metabolomics analytes fall below the limit of detection (LOD). If not properly addressed, this may introduce bias in effect estimates. To address this, we conducted a simulation study to evaluate the performance of linear, Tobit, Cox, and logistic modeling in the presence of below-LOD measurements in genome-wide association studies. We identified the optimal strategy as a two-step Linear-Tobit scheme, including rapid screening with linear regression followed by refinement with Tobit regression to retrieve accurate effect estimates. This higher accuracy helps mitigate a 1.3-fold and 2.7-fold inflation in causal estimates in a Mendelian randomization (MR) study, which would otherwise be present with 50% and 90% values below LOD. Validation through case studies on estradiol and testosterone levels in the UK Biobank confirmed the simulation results across subgroups with varying proportions of below-LOD measurements. The Linear-Tobit scheme offers optimal detection power and efficiency, with a focus on its applicability to biobank-scale datasets and accuracy in effect estimates to mitigate bias in downstream applications such as MR and polygenic risk scores.

## Introduction

High-throughput technologies enable comprehensive measurements of genetic variation and molecular phenotypes, with a promise to facilitate the understanding of the molecular mechanism leading to disease and guide the development of new treatments and preventive methods. For example, over the past decade, technologies for large-scale analyses of plasma proteins have undergone rapid development, and the number of proteins that can be measured in a study has increased from below 100 [1] to several thousand per sample. The use of these large-scale technologies has also become increasingly prevalent. One example is in the UK Biobank (UKB), where the genotype data and initial 34 biochemistry markers [2] in all 500 000 participants have been extended with whole-genome sequencing and 249 metabolic measures using the Nightingale Health NMR platform [3]. Additionally, 2941 protein profiles covering 54 219 participants [4] have been generated using the Olink Explore Platform as part of the UK Biobank Pharma Proteomics Project (UKB-PPP).

The growing production and availability of biomarkers and various omics data along with the emergence of large-scale cohorts provide promising potential for enhancing the characterization of disease pathways and identifying novel biomarkers. However, most high-throughput multiplexed methods come with the caveat that analytical sensitivities vary across features, some of which could have levels below the technical limit of detection (LOD) for a large fraction of samples. Even if those measurements are provided, such as in the proteomic and metabolomic data of the UKB, the demand for judicious interpretation is acknowledged by previous studies [5]. In the UKB proteomic dataset, for example, 657 (22.3%) protein analytes have >50% of samples below LOD, among which 500 have >70% [4].

Statistically, values below the LOD can be referred to as latent measurements, as they represent levels too low to be reliably quantified. These values may represent levels that are absent altogether, but more often, are undetectable due to limitations of the assay’s detection capabilities. This uncertainty and the resulting biased data distribution were less of an issue in traditional single-biomarker studies or for diagnostic tests, since they often focused on detecting extremely elevated outliers outside the normal range. For example, BNP (B-type natriuretic peptide) [6], a marker of heart disease, is relatively rarely detected in healthy young or middle-aged individuals but dramatically increases in persons with heart disease. In contrast, omics-related studies nowadays often make use of more delicate measurement scales, such as identifying genetic determinants of the between-individual variations in the levels of different molecules as well as discovering specific varying molecular levels, or molecular signatures, that serve as biomarkers for diagnosis or prediction for disease risk or progression. The complexity of analyses has increased, but there is still a lack of extensive biostatistical considerations, where modeling functions are very important for uncovering model assumptions and data distributions.

Traditionally, latent measurements have often been excluded, i.e. truncated, from data analyses [1, 7–9]. This can lead to selection bias, decreased sample sizes, and reduced power in detecting disease-related effects or genetic associations. For example, a homozygous genotype that causes a premature stop codon may result in the complete absence of a specific protein measurement by a given assay. Individuals with such genotype will then be excluded, and the genetic marker might even be filtered out due to deviation from Hardy–Weinberg equilibrium (HWE). In some recent studies, in attempting to increase the detection power for genetic signals in genome-wide association studies (GWAS), the latent measurements have instead been included upon availability without additional preprocessing for the potentially introduced noise [4, 10]. Whether to retain the latent values or not relies on the magnitude of noise that deviates the measurements, which varies across studies, yet this tradeoff has not been addressed in previous studies.

It should be noted that, to compromise with the increasing data and analytical complexity, there are numerous machine-learning-based approaches for handling latent values available, such as the K-nearest neighbor (KNN) and Monte Carlo methods for imputing missing data [11–13]. However, such methods are mostly used when the omics data are explanatory variables and while the analytical tools do not allow missing explanatory data. Thus, they are less suited for GWAS, where the omics traits are the outcome variable.

For handling latent (below-LOD) measurements in outcome variable, a straightforward approach is assigning them to a designated value such as LOD, LOD/2, or LOD/$\sqrt 2 $ [14, 15], in which substitution with the LOD is referred to as censoring. Usually, to ensure the assumption of normal distribution, rank-based inverse normal transformation is often applied [16] for the data to be analyzed using linear regression, which is supported by all GWAS software developed to handle large, biobank-sized cohorts, such as PLINK2 [17], SAIGE [18], and REGENIE [19]. While this is a commonly applied approach, it is unclear how the accuracy of effect estimation and statistical power depend on the fraction of latent measurements. With a large proportion of latent measurements, some studies dichotomize the quantitative phenotypes into binary categories (below- or above-LOD) and analyze them with logistic regression [9, 20], which is also supported by most GWAS software.

Another relatively rarely used approach in GWAS is the Tobit regression, which is specifically designed for modeling linear relationships when the prediction variable is censored [21]. However, its computational complexity limits the practicability for biobank-sized cohorts. Previous genetic studies using the Tobit model have either involved relatively small sample sizes [16, 22] or used it only for refining estimates of the significant variants already identified [20]. Likewise, although some non-Gaussian models for semicontinuous data, such as the negative binomial and compound Poisson–gamma, were adopted too, and the latter has been suggested for higher robustness [16], they lack evaluation regarding efficiency and are more sensitive to model misspecification. Instead, Cox proportional hazards (PH) model, which has the potential to incorporate both quantitative phenotypes and censoring status, does not rely on specific assumptions on data distribution [23]. Recently developed tools, such as COXMEG [24], GATE [25], and ADuLT [26], have made the analyses with Cox PH models feasible for biobank-scale GWAS. However, the validity of applying the Cox PH model to biomarker data with latent measurement—akin to right-censoring in survival analysis—has not been thoroughly evaluated yet.

With the lack of consensus on how to model omics data with latent measurements in GWAS, in combination with the increasing number of large-scale studies and availability of multi-omics datasets, there is a significant need to systematically revisit this topic to direct the optimal strategies for analyzing such data. We therefore present a comprehensive simulation study comparing different models—linear, Tobit, Cox, and logistic—in GWAS and propose a two-step approach, the Linear-Tobit scheme, as the optimal method for large-scale biobank studies. The assessments concentrated on the key outcome characteristics relevant to GWAS, including the detection sensitivity, computational efficiency, and accuracy in effect estimates. The latter is emerging as an essential and highly significant aspect with the growing interest in leveraging GWAS summary statistics for deriving polygenic risk scores (PRS) and Mendelian randomization (MR), where an accurate effect estimate is crucial. To validate the effectiveness of the different modeling strategies, we also conducted three GWAS case studies on biomarkers with different proportions of latent measurements in the UKB datasets.

## Materials and methods

### Simulated data generation

**Figure 1.**
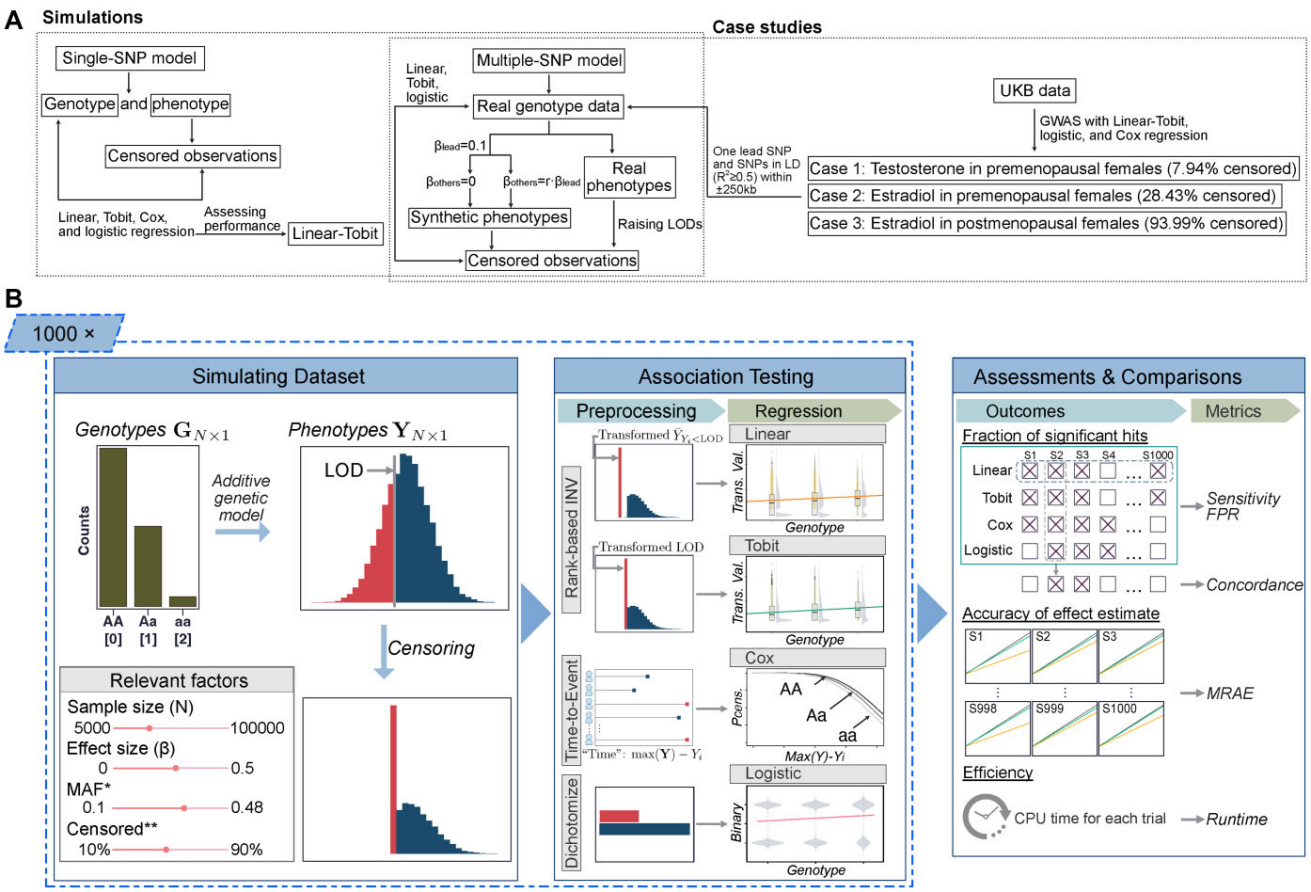
Study overview and workflow of the single-SNP baseline simulation and analysis. (**A**) The study consists of using purely simulated data under a single-SNP model for initial inspection of model performance, a series of case studies with UKB data to test the models and validate the simulations in real GWAS, and for further validation, particularly about the detection of causal variants and the correlated ones, we extracted one lead SNP and its nearby SNPs from case studies and tested the regression models with both totally simulated phenotypes and partly modified phenotypes based on real data. (**B**) We used a single-SNP model to capture the impact of varying factors on GWAS model performance. Datasets of genotypes (G) and phenotypes (Y) for each parameter set were prepared 1000 times following the principles: Genotypes G of N individuals were generated with the counts of each genotype based on the minor allele frequency (MAF) under the HWE; the phenotypes prior to censoring were formulated using the additive genetic model, linearly associated with the genotypes with an effect size $\beta $. The linear, Tobit, Cox, and logistic models were applied for regression against the preprocessed phenotype values and compared based on the five metrics, where the mean relative absolute error (MRAE) was computed only for the linear and Tobit models. Abbreviations: MAF—minor allele frequency; FPR—false positive rate; MRAE—mean relative absolute error; LD—linkage disequilibrium. Notes: *MAF: $( {2{{n}_2} + {{n}_1}} )/( {2N} )$, where ${{n}_1}$, ${{n}_2}$ are the counts of heterozygous (Aa) and minor (aa) alleles, respectively; **Censored: ${{P}_{{\mathrm{censored}}}} = P( {Y < LOD} )$.

We implemented two types of simulation designs (Fig. 1A). One is purely simulated, in which both genotypes and phenotypes were generated under a single-SNP (single nucleotide polymorphism) model to evaluate baseline model performance. The other is hybrid, which combined real genotype data with phenotypes that were both simulated and derived from real measurements with modified LODs, to further assess the methods under realistic genetic architecture reflecting linkage disequilibrium (LD) among variants.

With purely simulated data, model performance of identifying SNPs was evaluated across different population sizes, proportions of censored (below-LOD) measurements, MAFs, and SNP effect sizes. We generated genotype and phenotype datasets for populations ranging from 5000 to 100 000 individuals. For each scenario, 1000 datasets were simulated following the procedure outlined below and illustrated in the pipeline shown in Fig. 1B.

#### Genotypes

SNPs were simulated under the assumption of HWE. For each SNP, alleles were generated based on the predefined MAF, from 0.1 to 0.48, ensuring that the genotype proportions adhered to the expected HWE $\sim Binomial( {2,q} )$: with $p$ and $q$ representing the major and minor alleles, respectively, ${{p}^2}$ becomes the proportion for the homozygous major (encoded as “0”), $2pq$ for heterozygous (“1”), and ${{q}^2}$ for the homozygous minor (“2”); thus, with $q$ being the MAF, in a total sample size N, the expected genotype counts are ${{n}_0} = N{{p}^2}$, ${{n}_1} = N \cdot 2pq$, and ${{n}_2} = N{{q}^2}$.

#### Phenotypes

The genetic variants were associated with the outcome, which in this case is the biomarker measurements, under the assumption of additive allele effects as the linear relation $y = \beta \cdot g + \epsilon $, where $\beta $ ranging from 0.025 to 0.5 is the effect size, $g$ represents the genotype, and $\epsilon \sim \mathcal{N}( {0,1} )$ is the random noise. In the tests for Type I error, $\beta = 0$. We introduced left-censoring to the normally distributed phenotypic values by setting thresholds at the 10th, 30th, 50th, 70th, and 90th percentiles of the data. In each scenario, values below the respective threshold were replaced with the threshold value, resulting in 10% to 90% of the data being censored. The initial observations of phenotypes were thus recorded as ${{y}_{obs}} = max( {y,\ {\mathrm{LOD}}} )$. As described below, the censored values (${{y}_{obs}} = {\mathrm{LOD}}$) were further transformed depending on the specific model used for the analyses. We also conducted baseline simulations with uncensored samples.

In the hybrid setup, genotype data were extracted from regions within ± 250 kb of one of the lead SNPs (rs858516) identified in the subsequent UKB case studies. Variants within the region were selected based on LD, measured by squared Pearson’s correlation, ${{R}^2}$, to mimic a realistic GWAS scenario with correlated variants within a locus. We sorted the variants into high (${{R}^2} \ge 0.8$) and medium ($0.8 > {{R}^2} \ge 0.5$) LD groups, ending up with 6 in each. Two strategies were applied to simulate SNP effect sizes. The first assigned a non-zero effect ($\beta = 0.1$) only to the lead SNP, representing the scenario with a single causal variant. The second scaled effect sizes proportionally to their signed correlation, $R$, with the lead SNP, reflecting the signal distribution due to LD. Both strategies modeled the phenotypes using the additive assumption, with a sample size of 10 000, drawn 1000 times randomly from the participants of the case study to generate 1000 simulations. The phenotypes were also censored from proportions of 10% to 90%. Beyond the simulated phenotypes, we further used the real phenotype data from the UKB. Building on the case studies described later, the LOD was manually altered to introduce varying degrees of censoring so that we examined how increasing censored proportions affect the detection of associated variants under realistic measurement constraints. Although interactions (gene–gene or gene-environment) are known to occur in genetic architectures, their contribution to trait variance beyond additive effects is relatively sparse and hard to detect in GWAS [27, 28]; our simulations did not incorporate interaction terms.

### Statistical methods

In the context of GWAS, aimed at identifying SNPs associated with biomarker levels, we selected four models: the linear model, Tobit model, Cox proportional hazards model, and logistic model. Our single-SNP simulation used all four models, while the hybrid only used the linear, Tobit, and logistic, as the hybrid simulations were conducted after some validations on methods and the case studies. These models were chosen after careful consideration of factors such as ease of implementation in terms of the availability of existing GWAS tools and their suitability for handling different types of outcomes. Additionally, the data preprocessing strategies varied by model to accommodate their specific assumptions and requirements.

#### Linear model

The biomarker levels were modeled using the following linear equation ${{y}^{\mathrm{*}}} = {\mathrm{\beta }}_0^* + {{{\mathrm{\beta }}}_1}{{}^*}g + {{\epsilon }^{\mathrm{*}}}$, where ${\mathrm{\beta }}_0^*$ is the intercept, ${\mathrm{\beta }}_1^*$ denotes the SNP effect size, and the residual error term ${{\epsilon }^*}$ is assumed to be normally distributed ${{\epsilon }^*} \sim \mathcal{N}( {0,{{{\mathrm{\sigma }}}^2}} ).$ Despite the simulated phenotypes being normally distributed, we applied an inverse normal rank-based transformation to align with standard practices in GWAS analysis. This preprocessing step was performed using the RNOmni [29] R package.

To account for latent values below LOD, we explored two approaches. Initially we began with the most straightforward approach commonly used in previous GWAS studies: conducting linear regression using only the observable measurements. All latent values were removed, and rank transformation was solely applied to the above-LOD data, with the aim of mitigating the influence of censored values. Although as expected the results from such significantly reduced sample size were not promising (Supplementary Fig. S1B), this step gave quick insight into the extent that data truncation could impact test outcomes.

For the full dataset, we addressed latent, i.e. censored, values by imputing with the average of the rank-transformed values corresponding to those below the LOD, using RankNorm function in the RNOmni [29] R package with parameter ties.method set to “average.” This option assigns each tied value, which in this case is the censored, to the average rank of that group. The linear regression was performed by the lm() function from the stats [30] R package.

#### Tobit model

The observed variable ${{y}_{obs}}$ is treated as left-censored representation of an underlying normally distributed latent variable ${{y}^*}$, which follows the linear equation described above, such that


\begin{eqnarray*}
{{y}_{obs}} = \left\{ {\begin{array}{@{}*{1}{c}@{}} {{{y}^{\mathrm{*}}}{\mathrm{\ if\ }}{{y}_{obs}} > {\mathrm{LOD}}}\\ {{\mathrm{LOD}},{\mathrm{\ otherwise.}}} \end{array}} \right.
\end{eqnarray*}

The Tobit model thus makes estimations using likelihood contributions from the above-LOD values and the probability of being censored. Although it inherently accounts for censored observations through its likelihood function, we still applied rank transformation to all observations, not just for ensuring consistency of the phenotype scale across models but also for the fact that biomarker distributions are often skewed. Unlike for the linear model, here we set ties.method to “max” during the usage of RankNorm function from RNOmni [29]. This option assigns censored observations the largest rank within their tied group, corresponding to those at the smallest uncensored measurement. Tobit regression was implemented by the censReg [31] R package.

We used different tie-handling methods for the linear model to align with the underlying assumptions, where “average” is to preserve a central, less biased tendency among tied values for the linear model and “max” is used to account for left-censoring and prioritize the lower limit of truncated effects. Despite the usage being intuitive, to justify the tie-handling strategies, a brief simulation with 500 runs of linear and Tobit regression against $y$ on data of N = 1000, $x \sim \mathcal{N}( {0,\ 1} )$, $\beta = 0.1$, $y = \beta \cdot x + \mathcal{N}( {0,\ 1} )$, with censored proportions set to 10% ∼ 90%, showed that setting ties.method=“max” for the linear model caused more underestimation of the effect sizes and thus less significance than “average,” which gave the estimation closer to the simulated slope, while Tobit model always had estimation closest to the true (Supplementary Fig. S2).

#### Cox model

Although the Cox model is widely used for analyzing survival or time-to-event data, Dinse *et al.* proposed an approach that treats the LOD exposure variable as a censored outcome [23]. The first step transforms the left-censored measurements into right-censored data by defining ${{y}_{cox,i}} = max( {{{y}_{obs}}} ) - {{y}_{obs,i}}$, as to mimic a “time” scale. The second step integrates the quantitative data with the censoring status, treating above-LOD as “events,” i.e. ${{\delta }_i} = \mathbb{I}( {{{y}_{{\mathrm{obs}},i}} > {\mathrm{LOD}}} )$, and defines the function as follows:


\begin{eqnarray*}
h\left( {{{y}_{cox}}{\mathrm{|}}g} \right) = {{h}_0}\left( {{{y}_{cox}}} \right)\exp \left( {{{{\mathrm{\beta }}}^{\mathrm{*}}} \cdot g} \right),
\end{eqnarray*}

where $h( {{{y}_{cox}}{\mathrm{|}}g} )$ is the hazard function at the transformed measure ${{y}_{cox}}$ given the covariate, or SNP $g$ in this single-gene setting, ${{h}_0}( {{{y}_{cox}}} )$ is the baseline hazard function representing the hazard with the non-effect allele, and ${{{\mathrm{\beta }}}^{\mathrm{*}}}$ is the coefficient estimate, which denotes the change in the log hazard rate per effect allele. As it does not require assumptions about the underlying distribution of quantitative measures, we did not perform the rank transformation for the measurements. The Cox regression model was fitted with the transformed measure ${{y}_{cox}}$ and the censoring indicator $\delta $ as inputs.

However, while the approach is appealing for its flexibility and ability to handle both quantitative and qualitative features, it carries the risk of violating the PH assumption [32]. The assumption requires that the effect of a predictor, such as the SNP $g$, on the hazard remains constant over “time,” represented by the transformed measure ${{y}_{cox}}$, which our objective to test this association appears to contradict. Although the model remains operational, we had concerns about its validity and performed the Schoenfeld residuals test to examine potential violations of the PH assumption. Thresholds for the Schoenfeld test were set at 0.05 and 5e^−8^, the latter being a commonly used significance threshold in GWAS, to assess the extent and severity of potential violations of the assumption. Both the model fitting and assumption testing were conducted using the survival [33] R package.

#### Logistic model

In the presence of LOD, biomarker levels can be classified as a binary outcome, either below or above the LOD, like the censored indicator $\delta $ described earlier. The model predicts the probability that that $\delta = 1$ given the predictor $g$, forming a linear combination of the SNP to impact the log odds as


\begin{eqnarray*}
\log \left( {\frac{{{{P}_{{\mathrm{\delta }} = 1}}}}{{1 - {{P}_{{\mathrm{\delta }} = 1}}}}} \right) = {\mathrm{\beta }}_0^{\mathrm{*}} + {\mathrm{\beta }}_1^{\mathrm{*}}g.
\end{eqnarray*}

The coefficient ${\mathrm{\beta }}_1^{\mathrm{*}}$ represents the log odds of being above the LOD per effect allele, providing insights into the factors associated with detectable or undetectable levels. The logistic model was implemented by the glm() function from the stats [30] R package.

### Model comparison

The large number of simulation trials provided sufficient precision in evaluation metrics, robustness against random noise in the estimations, and comprehensive coverage of parameter settings. We assessed model performance based on several key criteria to identify the most robust strategy.

#### Sensitivity and FPR

Computing the detection rate, i.e. fraction of trials with *P*-value less than the significance cutoff out of the corresponding 1000 simulations, gave the true positive rate (TPR) from $\beta \not= 0$ trials and false positive rate (FPR) from $\beta = 0$ trials. Referred to as sensitivity, the TPR measures the likelihood of successfully detecting genetic associations, with the *P*-value of 5e^−8^. Even though the simulation was modeled with only one SNP, we chose this commonly used significance threshold for consistency with GWAS routine, ensuring the comparability between our study and other GWAS analyses. The strict standard also enhanced the clarity for observing the trend in model sensitivity as the parameters varied. For FPR, however, we first set the *P*-value cutoff to be 0.05 mainly for visual clarity followed by 1e^−5^, commonly used as the “suggestive association,” because the strict threshold 5e^−8^ would result in FPR = 0 for all models and obscure potential differences.

We used the first threshold *P*= 0.05 for preliminary computational verification of the simulation setups. With 1000 simulations, the distribution of FPR follows $Binomial( {1000,0.05} )$, of which the Normal (Wald) 95% confidence interval is (0.0365, 0.0635). Thus, before running the simulations with non-zero effect sizes, we checked whether the models give FPRs within the expected intervals.

#### Concordance rate

Scenarios with moderate sensitivity raised the question of whether all models were (un)able to detect the association. We computed the overall and pairwise concordance rate as the number of trials in which the models agreed on testing significance out of the corresponding 1000 simulations. The higher the concordance rate with the most statistically powerful model, the more reliable a model was expected to be. Since the linear model demonstrated the highest sensitivity, we used it as the reference point for comparison. Pairwise comparisons also revealed that the logistic model was most often the only one in strong disagreement with the others. Hence, we assessed each model’s concordance with the linear model for consistency and convenience.

#### MRAE

Since a linear relationship was assumed for simulating the biomarker levels, we evaluated the accuracy of the linear and Tobit models by calculating the mean relative absolute error (MRAE) in their effect estimates ${{\beta }^*}$. For each scenario,


\begin{eqnarray*}
{\mathrm{MRAE}} = \frac{1}{{{{n}_{{\mathrm{sim}}}}}}\mathop \sum \limits_{i = 1}^{{{n}_{{\mathrm{sim}}}}} \left| {\frac{{{\mathrm{\ }}\beta _i^* - {{{\mathrm{\beta }}}_{\mathrm{i}}}}}{{{{{\mathrm{\beta }}}_{\mathrm{i}}}}}} \right|,
\end{eqnarray*}

where ${{{\mathrm{n}}}_{{\mathrm{sim}}}} = 1000$ denotes the number of simulations, ${{{\mathrm{\beta }}}_{\mathrm{i}}}$ the true effect, and $\beta _i^*$ the effect estimates by the corresponding model for the $i$-th simulation.

#### Computational efficiency

To assess scalability in practice, we benchmarked the runtime of each model. The time from model initialization to convergence was measured for each trial using the R function proc.time(), and the results were averaged for each scenario.

### Potential inflation in MR Wald ratio estimator

For an illustrative example of the consequence of biased effect size, we deduced the theoretical inflation of the MR Wald ratio estimator $\beta _{{\mathrm{MR}}}^* = \beta _{{\mathrm{GO}}}^*/\beta _{{\mathrm{GE}}}^*$, where $\beta _{{\mathrm{GO}}}^*$ and $\beta _{{\mathrm{GE}}}^*$ are genetic variant-outcome and -exposure associations. For simplicity, the instrument, i.e. genetic variant, $G$ is assumed to be strongly associated with the exposure $E$, and independence is assumed between the estimators $\beta _{{\mathrm{GO}}}^*$ and $\beta _{{\mathrm{GE}}}^*$. Thus, the expected Wald ratio estimator could be approximated by


\begin{eqnarray*}
\mathbb{E}\left( {\beta _{{\mathrm{MR}}}^{\mathrm{*}}} \right) = \mathbb{E}\left( {\frac{{\beta _{{\mathrm{GO}}}^{\mathrm{*}}}}{{\beta _{{\mathrm{GE}}}^{\mathrm{*}}}}} \right) \simeq \frac{{\mathbb{E}\left( {\beta _{{\mathrm{GO}}}^{\mathrm{*}}} \right)}}{{\mathbb{E}\left( {\beta _{{\mathrm{GE}}}^{\mathrm{*}}} \right)}} = \frac{{{{\beta }_{{\mathrm{GO}}}}}}{{\left( {1 + \eta } \right){{\beta }_{{\mathrm{GE}}}}}},
\end{eqnarray*}

where ${{\beta }_{{\mathrm{GO}}}}$ and ${{\beta }_{{\mathrm{GE}}}}$ are the true effects of the genetic variant $G$ on the outcome $O$ and exposure $E$, respectively. Here, the expectation of $\beta _{{\mathrm{GO}}}^*$ is assumed to be unbiased such that $\mathbb{E}( {\beta _{{\mathrm{GO}}}^*} ) - {{\beta }_{{\mathrm{GO}}}} = 0$, while $\eta $ denotes the relative attenuation bias, in the effect on $E\ $estimated by the linear model due to the presence of censored measurements, such that


\begin{eqnarray*}
\eta {\mathrm{\ }} = \frac{{\mathbb{E}\left( {\beta _{{\mathrm{GE}}}^{\mathrm{*}}} \right) - {{\beta }_{{\mathrm{GE}}}}}}{{{{\beta }_{{\mathrm{GE}}}}}}.
\end{eqnarray*}

Since a systematic underestimation was observed, $\eta < 0$, and the inflation factor becomes


\begin{eqnarray*}
\frac{{\mathbb{E}\left( {\beta _{{\mathrm{MR}}}^{\mathrm{*}}} \right)}}{{{{\beta }_{{\mathrm{MR}}}}}} = {{\left( {1 + \eta } \right)}^{ - 1}}.
\end{eqnarray*}

Aligning with the simulations where the impact of censored proportion was the focus, we thus estimated the relative bias in the effect estimates, $\hat{\eta }$, of each censored proportion as


\begin{eqnarray*}
\hat{\eta } = \frac{{\mathbb{E}\left( {\beta _i^{\mathrm{*}}} \right) - {{{\mathrm{\beta }}}_{\mathrm{i}}}}}{{{{{\mathrm{\beta }}}_{\mathrm{i}}}}} = \frac{1}{{{{n}_{\textit{scenarios}}}}}\mathop \sum \limits_i^{{{n}_{\textit{scenarios}}}} \frac{{\beta _i^{\mathrm{*}} - \beta_i}}{{\beta_i}},
\end{eqnarray*}

in which ${{n}_{\textit{scenarios}}} = {{n}_{sim}} \cdot {{n}_{\textit{Sample}\ \textit{Size}}} \cdot {{n}_{\textit{Effect}\ \textit{Size}}} \cdot {{n}_{MAF}}$ represents all scenario sets with the varying factor values stated above for the corresponding censored proportions.

### Computational environment

The computations of simulations and the following case studies were performed with 16 cores, 8 GB of memory each, on Bianca, the high-performance computing (HPC) cluster dedicated for sensitive data analyses on Uppsala Multidiciplinary Center for Advanced Computational Science (UPPMAX).

### UKB case studies

The UKB is a large-scale biomedical database including genetic, health, and lifestyle information covering ∼500 000 participants, under the ethics permit REC [ref. 11]/NW/0382. Currently it features 34 biomarkers, with more proteomics and metabolomics data becoming available. In our parallel project involving hormones, especially estradiol levels (UKB Project No. 41143), we observed a large proportion of censoring in certain population groups. The stratification process is shown in Supplementary Fig. S8. Unrelated participants and those with genetic relatedness factor $ \le $0.044 (Field 22011.0.0; Field 22012.0.0) with Caucasian ancestry (Field 22006.0.0) were included; those who were receiving hormonal replacement therapy (Field 2814.0.0) were excluded. To obtain a gradient of censored proportion as in the simulation study, we used the measured concentrations of testosterone (Field 30850.0.0 & 30856.0.0) in premenopausal females (Case 1) and estradiol (Field 30800.0.0 & 30806.0.0) in premenopausal (Case 2) and postmenopausal (Case 3) females. Ethical approval for the analyses of this study was approved by the Swedish Ethical Review Authority (Dnr: *2020-04415*).

### Example GWAS for comparing modeling approaches

With the imputed genotype data containing 93 093 070 SNPs from the release v3, we only analyzed those with MAF > 0.01, HWE *P*-value > 1e^−20^, and missingness <5%. The RNOmni [29] R package was used to perform rank-based inverse normal transformation on the measurements following rules stated in previous sections. We conducted GWAS with the linear and logistic models using PLINK2.00-alpha-3.7-20221024 [17], and we used GATE V.0.45 [25] for the Cox-model approach. The first step of GATE [25] involved the computation of a full genetic relatedness matrix (GRM). For Case 1 and Case 2, 262 356 pruned (500 kb window, sliding step-size 50 markers, ${{r}^2}$ < 0.2) genotyped markers with MAF ≥ 0.01 were used, in alignment with the criteria used by the developer of GATE [25]. For Case 3, due to the larger sample size, we adopted the 71 228 markers with SNP-load <0.003 for the top three principal components (PCs) and MAF ≥ 0.01, the criteria used initially by Bycroft *et al.* for estimating kinship coefficients [34]. All analyses were adjusted for covariates including age (Field 21003.0.0), BMI (Field 21001.0.0), smoking habits (Field 20116.0.0, 1249.0.0, 1239.0.0), surgical history (bilateral oophorectomy and hysterectomy, Field 2834.0.0, 3591.0.0, 20004.0.0), genotyping batches (Field 22000.0.0), and the first 15 PCs (Field 22009.0.1–22009.0.15). For the premenopausal group, menstrual status (Field 3720.0.0) and oral contraceptive use (Field 2804.0.0) were also included as covariates.

The clump function in PLINK2 [17] was applied to extract independent lead variants from the GWAS, in which the main parameters were set to ${{p}_1}$= 5e^−8^, ${{p}_2}$= 1e^−4^, and ${{R}^2}$= 0.1 with the window size of $kb$= 1000. The Manhattan and Q–Q plots for visualizing GWAS results were generated by the qqman [35] R package. The extracted variants were further analyzed with Tobit model by censReg [31], which was combined with the screening with linear regression by PLINK2 [17] into censGWAS, an R package, as a convenient implementation for the Linear-Tobit scheme.

## Results

### Systematically assessing GWAS regression models via simulations

The simulation study involved varying latent proportions in hypothetical biomarker/omics phenotype measurements, set between 10% and 90% with increments of 20% (Fig. 1). For each setup, 1000 simulations were performed with the four different models—linear, Tobit, Cox, and logistic models—each with a specific data preprocessing step. The results from simulations were then used to calculate the performance metrics. For clarification, quantitative traits were simulated, and the measurements below a designated threshold, i.e. an artificial LOD, were censored. The threshold values were chosen to create varying proportions of censored values. In the simulation study, we therefore use the term “censored proportion,” and the values below the artificial LOD are “censored values.” Our survey across a thorough range of scenarios aimed to identify the optimal regression model(s) for biobank-scale GWAS. Meanwhile, our results visualized trends in model performance, reflecting the combined impact of the sample size along with the effect size and MAF of the genetic variant, which are the factors most relevant to genetic association tests (Fig. 1 and Supplementary Fig. S1A). To verify the simulation setup before proceeding to performance evaluation, we also checked that the FPR resulted from trials with zero effect size aligned with the expected quantiles (Supplementary Fig. S3A).

### Testing bias arising from censored phenotypes

Baseline simulations, without any censored values, showed that a small sample size (N < 10 000) could be underpowered to detect a genetic variant with an effect size <0.05, considering a high sensitivity as successfully detecting a variant with at least 95% chance when the significance cutoff commonly used in GWAS, 5e^−8^, is applied (Supplementary Fig. S1A). Since no values were censored, the baseline tests were conducted using linear regression. The reduced sensitivity observed in subsequent simulations should not solely be attributed to the censored proportion, instead. It also reflected computational constraints of the built-in algorithm, such as numerical instability and precision limits. As expected, we observed strongly decreasing sensitivity with the reduced sample size that resulted from truncating observations below the artificial LOD (Supplementary Fig. S1B).

**Figure 2.**
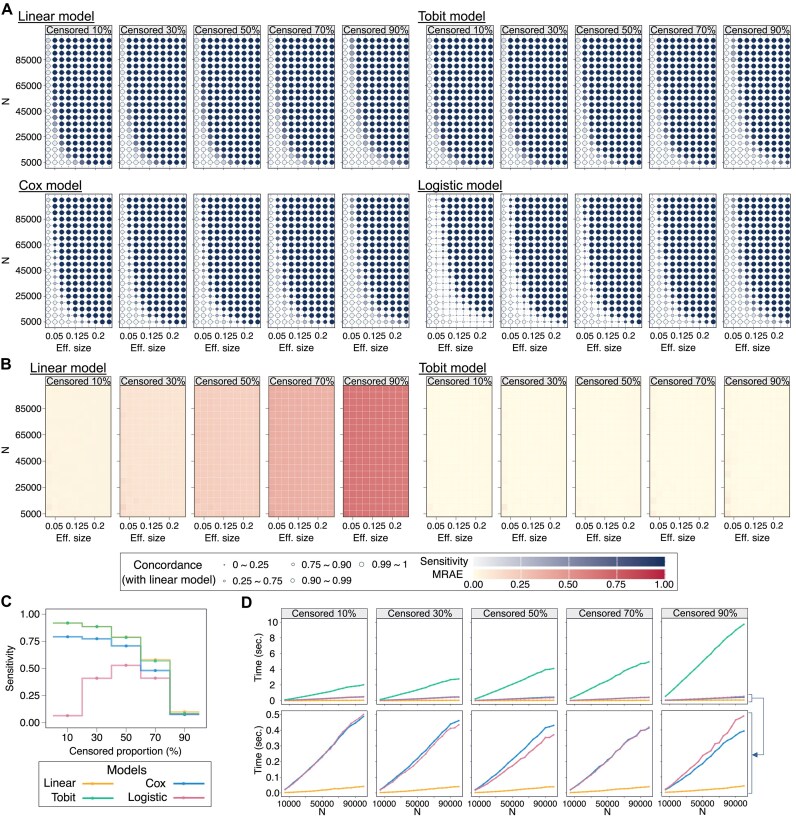
Model performance of simulated GWAS analyses. In the demonstration of model sensitivity (**A**) and estimation accuracy (**B**) regarding the varying sample size (N, 5000–10 000) and effect size (Eff. size, 0.025–0.25), the MAF of the simulation genotypes is fixed to 0.2. The tint of each bubble/block indicates the magnitude of in the resulting sensitivity and mean relative absolute error (MRAE) of the corresponding scenario; the size of bubble represents the concordance rate of the other three models to the linear model. The step function showing the sensitivity of each model regarding the censored proportion (**C**) is drawn from the scenario with N = 10 000, Eff. size = 0.1, and MAF = 0.4. The runtime (**D**) is averaged over the 1000 simulations under each varying N with MAF = 0.2 and Eff. size = 0.1 the figures in the second row of (D) represent the zoomed-in portion of the first row.

We showcased the sensitivity of the four tested models with varying sample size and effect size under different censored proportions (Fig. 2A). In the linear, Tobit, and Cox models, sensitivity dropped substantially when more than half of the measurements were censored (Fig. 2A), but the loss in sensitivity was mitigated compared to excluding latent values (Supplementary Fig. S1B). On the contrary, the logistic model showed decreased sensitivity as the censored proportion diverted from 50%, part of which could potentially be attributed to class imbalance. The logistic model consistently had the lowest sensitivity among the tested models across all scenarios, followed by the Cox model, while the linear and Tobit models always had the highest. The largest difference in performance occurred with the lowest censoring proportion, but with 90% censored all models performed almost equally (Fig. 2C). We also observed lower concordance rate in the Cox and logistic models, especially the latter, with the linear model (Fig. 2A). The same conclusions were made regarding the varying MAF, which had the least impact on model performance compared to the other factors (Supplementary Figs S4A and S5A). Interestingly, there was also a tendency of an increase in the FPR with the fraction of censored measurements, as the bias in estimated effect size could bring misestimated *P*-values, though not as substantial as the decrease in sensitivity (Supplementary Fig. S3B).

Given that the linear and Tobit models showed higher sensitivity than the others, we limited our assessments of accuracy in the effect estimate to these two (Fig. 2B and Supplementary Figs S4B and S5B). The MRAE in the effect estimates for the linear model increased with the censored proportion, to which the error grew at an accelerating rate. Furthermore, examining signed errors instead of absolute errors revealed systematic underestimation of the “true” effect by the linear model. For censored proportions of 10%, 30%, 50%, 70%, and 90%, averaging the errors across all corresponding scenarios showed that it has underestimated the effects by ~4.23%, 11.9%, 22%, 36.5%, and 63.3%, respectively. In contrast, the Tobit model, specifically designed for handling censored data, remained more accurate, with the relative error of ~1.3%, almost unaffected by increased censoring. Also, the impact of censoring outweighed other factors in determining the accuracy.

We therefore suggest that the Tobit model should be used to recalibrate the effect estimate, even with censoring as few as 10% censored measurements, if the estimates are being used for downstream analyses such as MR and PRS, or for meta-analysis or multi-cohort comparisons where an accurate estimate is crucial. The attenuation bias in MR estimate, typically the Wald ratio estimator $\beta _{{\mathrm{MR}}}^* = \beta _{{\mathrm{GO}}}^*/\beta _{{\mathrm{GE}}}^*$ where $\beta _{{\mathrm{GO}}}^*$ and $\beta _{{\mathrm{GE}}}^*$ represent genetic variant-outcome and -exposure associations, respectively, was used for a quick illustration. As the biomarker measurements are usually used as the exposure, we deduced that theoretically the relative bias $\eta $ in $\beta _{{\mathrm{GE}}}^*$ would impact the causal estimate $\beta _{{\mathrm{MR}}}^*$ by a factor of ${{( {1 + {\mathrm{\eta }}} )}^{ - 1}}$, where $\eta < 0$ given underestimation. Based on the underestimated effect sizes by the linear model described earlier for the increasing gradient of censored proportions, the corresponding MR estimates could be elevated by 1.04-, 1.14-, 1.28-, 1.58-, and 2.73-fold, respectively (Supplementary Fig. S6).

### A pitfall in the Cox approach

Surprisingly, the Cox model did not outperform the others in terms of sensitivity, which could have been anticipated due to its ability to incorporate both quantitative and binary information about traits, e.g. time-to-event and if an event (above-LOD) occurs, which could have given it a distinct advantage. However, the Schoenfeld residual tests, used to validate the PH assumption, indicated significant violations under most parameter configurations (Supplementary Fig. S7), as also pointed out in Ortega-Villa *et al.* even though their study involved the censored data differently [32]. Hence, we concluded that using the Cox model as accommodation of censored phenotypes was unsuitable for analyzing biomarkers with latent measurements, despite its advantage of not relying on assumptions about data distributions.

### Linear-Tobit scheme for optimal efficiency and detection quality

Benchmarking the execution time showed that the Tobit model was the least scalable as indicated by its heavy computational demand (Fig. 2D). In contrast, the linear model was the most efficient against the increasing sample size. While suspecting their influence on the efficient model convergence from the numerical aspects, neither the effect size nor the MAF of the variant showed significant correlation with the runtime (Supplementary Fig. S8). These additional tests still confirmed the increasing computational cost of the Tobit model along with the censored proportion. Large-scale analyses require not only accuracy but also efficiency; thus, full-scale Tobit regression becomes impractical. A two-step Linear-Tobit scheme, using linear regression for initial screening and Tobit regression to specifically refine effect estimates of the significant variants, could balance the computational efficiency and detection quality.

### Applying Linear-Tobit scheme on UKB data

We examined the Linear-Tobit scheme using UKB data on testosterone and estradiol levels, where varying proportions of latent measurements exist in different female strata depending on menopausal status (Supplementary Fig. S9 and  Supplementary Table S1). We selected testosterone levels in 42 532 premenopausal female participants with 7.94% latent measurements (Case 1), estradiol levels in 40 459 premenopausal female participants with 28.43% latent measurements (Case 2), and in 131 310 postmenopausal female participants with 93.99% latent measurements (Case 3). Additionally, to validate conclusions from the simulation study, we also performed GWAS using both the logistic and Cox models for the three cases, even though the usage of Cox model was concluded to be less suitable.

It should be noted that, although the Cox “approach” was run with GATE [25], the tool for time-to-event GWAS with frailty model, we keep the term Cox “model” just for narrative consistency. Although in Cases 2 and 3, the Cox model performed decently, also identifying some variants that were not initially found by the linear model (Supplementary Table S2), it should not be over-appraised. The seemingly enhanced performance might be attributed to the software design of GATE [25], an extended version of SAIGE [18] that incorporates more advanced numerical techniques than PLINK2 [17], which we used to run the linear and logistic models in case studies. Moreover, early deviation from the null was found only in the Q-Q plots of the Cox model for Case 1, along with its lower sensitivity than the linear model (Supplementary Fig. S10B; Supplementary Table S2).

**Figure 3.**
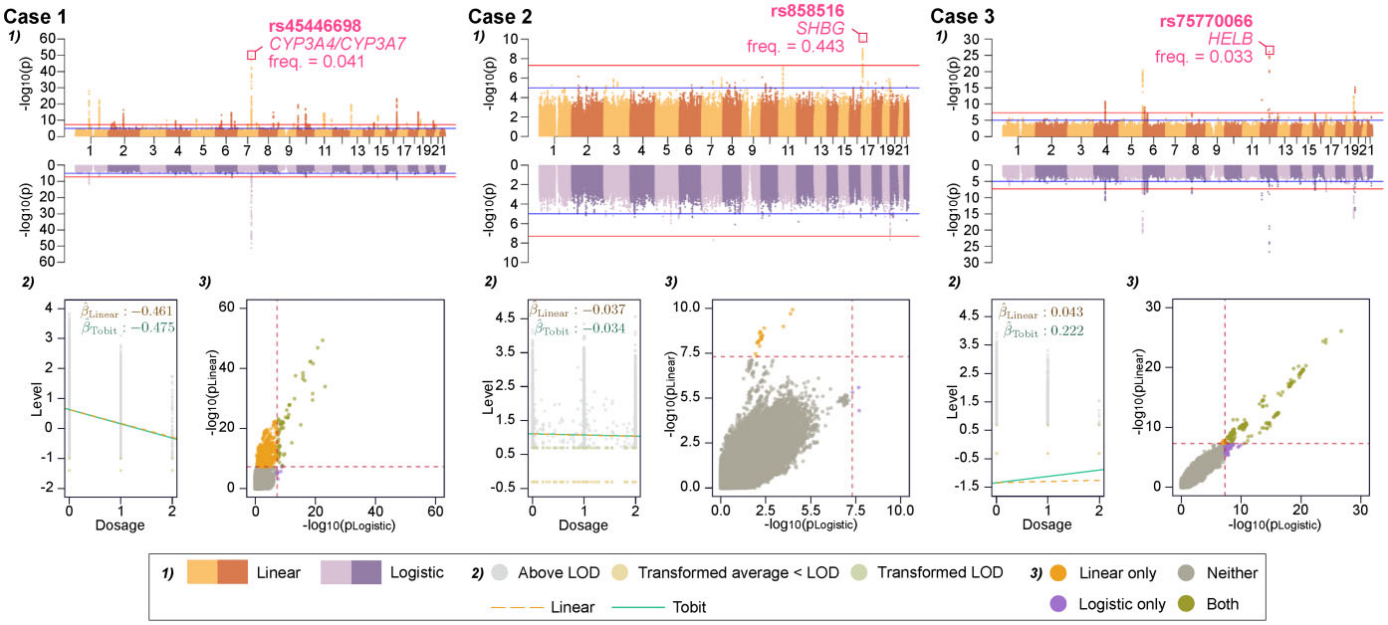
Example GWAS with UKB data validates the conclusions from simulation study. The cases are representative for each censored proportion in the corresponding biomarker (tested phenotype): Case 1 – testosterone in premenopausal female participants, N = 42 532, 7.94% censored; Case 2 – estradiol in premenopausal female participants, N = 40 459, 28.43% censored; Case 3 – estradiol in postmenopausal female participants, N = 131 310, 93.99% censored. For each case, 1) the Miami plot compares the GWAS outcome of the linear and logistic models, 2) the scatter plot with the regression line shows the refined effect estimate of the example hit, taken from the clumped GWAS results and highlighted in the Miami plots, by the Tobit model, and 3) the *P*-values of each model compare the significance, serving as indication of model concordance. Corresponding comparison with the Cox approach, despite not performing well in our simulations, and Q–Q plots of the GWAS results for all models are shown in Supplementary Fig. S10.

The initial screening with linear model indeed had the highest sensitivity overall (the largest number of independent significant GWAS hits, i.e. *P* < 5e^−8^) (Fig. 3 and Supplementary Fig. S10). In Case 1 (testosterone, 7.94% censoring rate), the linear model resulted in ~10 times as many significant hits as the Cox and logistic models, and all hits from the linear regression remained significant with Tobit (Supplementary Table S2). In Case 2 (estradiol in premenopausal females, 28.43% censoring rate), only four significant hits were identified (Supplementary Table S2). The concordance between the models was also low, and most findings were borderline significant. However, the most significant hit (rs858516, found by linear, Tobit, and Cox) mapped to the *SHBG* (sex hormone-binding globulin) locus, which is well-known to be associated with estradiol and its bioavailability. Notably, while the variants identified by the logistic could appear as potential false positives since neither of them were even close to being statistically significant in other models, one (rs34962991) is associated with *TMEM150B*, which has relevance to estradiol metabolism since it has previously been associated with menopausal age [36, 37]. In addition, the Cox model identified another variant (rs11031005) that was also significant in the Tobit but not in linear or logistic models (Supplementary Table S2). This variant is likely to be a true positive because of its location in the 5′-UTR of *FSHB* (follicle stimulating hormone subunit beta) locus, which is known for its role in direct stimulation of estradiol synthesis [38]. Despite the poor performance of the logistic model in the simulation, we could not rule out the possibility that it might have uniquely identified additional GWAS hits.

In Case 3 (estradiol in postmenopausal females, 93.99% censoring rate), we observed higher concordance among the models than in the previous cases, which agreed with the simulation study that higher censoring rate led to a higher concordance because of an overall decrease in sensitivity. Also, as concluded above, the linear model had systematically underestimated the effect size, making it always lower than those by Tobit; the estimates were the most evidently improved by the refinement using Tobit with this high censored proportion (Fig. 3 Case 3(2)). Nevertheless, it is worth noticing that there was slightly higher number of significant hits that resulted from the logistic model (12 hits) compared to the linear model (8 hits); 3 of the 4 hits had the significance asserted by the Tobit model (Supplementary Table S2). Such an increase in detection power observed with the logistic model did not align with the simulation study. We thus checked the distribution of the hormone levels of each genotype for all identified variants and observed that potential discrepancies in the model performances could occur when the quantitative measurements for a specific gene deviated from the assumption of additive genetic effect (Supplementary Table S3).

### LD-based simulations using genotypes around a lead SNP

As LD is considered a critical factor that potentially complicates GWAS findings [39], it is intuitive to think LD might affect model performance, thereby showing extra patterns besides the baseline simulations. To further validate the previous conclusions also when LD was considered, we extracted the genotype data around one lead SNP (rs858516) from Case 2. It had a more significant peak than the other loci of this case. More importantly, the case had a moderate censored proportion that also allowed for manually increasing the LOD to inspect a more realistic impact of censoring on detecting a likely causal SNP. However, with simulated phenotypes that ideally aligned with the normal assumptions and the modified estradiol levels that were skewed like most biomarker measurements, we did not observe patterns diverging from the previous conclusions.

We focused on the SNPs in LD with the lead within the window of ±250 kb, resulting in 6 SNPs (including the lead itself) with high LD (${{R}^2} \ge 0.8$) and 6 with medium LD ($0.5 \le {{R}^2} < 0.8$). In the GWAS example with the linear model, all 6 in the high LD group and one from the medium group reached the significance threshold of 5e^−8^ (Supplementary Fig. S11A). All of them mapped to the same genes as the lead. With multiple variants, we designed two ways of simulating the phenotypes as differed by how the effect sizes were defined. First, we assumed that only the lead SNP was causal and assigned it an effect size of 0.1, while the others had no effect (Strategy 1). Second, we had LD-proportional effects, where the SNPs except the lead had effects slightly smaller than 0.1, reflecting an LD-shaped polygenic scenario (Strategy 2). One thousand simulations were run with sample size of 10 000, in which the participants were randomly drawn from those of Case 2. The corresponding results are demonstrated in Supplementary Fig. S11C. Using the linear, Tobit, and logistic models, we again examined the sensitivity and concordance with the linear, which showed the same trend across the increasing censored proportions as in the single-SNP simulations. We did not observe any difference in sensitivities or concordance due to LD either. For both assumptions, all SNPs in the medium group had zero sensitivities along all censored proportions. To our expectation, all regression models experienced a milder decrease in sensitivity in Strategy 2 than Strategy 1, because the simulated effect size was closer to the marginal effect size, i.e. for a tag SNP $j$, $\widehat {{{{\mathrm{\beta }}}_j}} = {{{\mathrm{\beta }}}_{{\mathrm{lead}}}} \cdot R \cdot \sqrt {\frac{{{\mathrm{Var}}( {{{G}_{{\mathrm{lead}}}}} )}}{{{\mathrm{Var}}( {{{G}_j}} )}}} $.

To make additional validation, we modified the LOD in the corresponding estradiol measurements. Starting from the initial LOD of 175 pmol/l (28.43% censored), we raised it to 300, 500, and 900, resulting in censored proportions of 47.8%, 70.8%, and 89.1%, as Sets I–IV, respectively. The four sets of data thus reflected a spectrum of censored proportions. The Linear-Tobit scheme and the logistic model were used. For the linear model, we observe the linearly decreasing effect estimate along the increased censored proportions, where the detection failed for the SNPs in the high group in Set IV, and starting from Set III, the originally significant SNP from the medium group became undetected (Supplementary Fig. S11B). To maintain a visualization in linear scale, instead of the odds ratio, we directly used the log-odds and observed a different trend than the linear model, where the decrease in uncensored measurements did not always align to a decreased estimate (Supplementary Fig. S11B). But still, like in the single-SNP simulations where the logistic model got the highest sensitivity when the ratio between censored/uncensored became more balanced, i.e. closer to 50% censored, the lead SNP and those in high LD with it became detected in Sets II and III. Regardless of the LD structure, the SNPs experienced a comparable rate of underestimation by the linear model, while the Tobit estimates remained stable (Supplementary Fig. S11D). One may carefully notice that the decrease in the linear effect estimate was not linearly proportional to the LD, which is partly due to the difference in MAF resulting in the fraction of genetic variances between the lead and the tested, as the marginal effect shown in the formula above. However, since such difference was subtle compared to the Pearson’s correlation index and out of the scope of validating the simulation conclusions, we decided not to incorporate the genetic variance in our multi-SNP simulations.

To summarize, the consistency between these LD-based simulations and the single-SNP simulations suggests that LD did not alter the censoring-induced pattern of sensitivity or accuracy but primarily transmitted the same signal attenuation across correlated SNPs rather than introducing additional bias.

## Discussion

In this study, we show that a combined Linear-Tobit scheme is in general the optimal approach for performing biobank-scale GWAS on phenotypes with latent measurements that are below the LOD in assays. The increasing prevalence of such phenotypes, driven by advances in high-throughput technologies of omics data measurements, serves as a key motivation for addressing these challenges. While earlier studies [16, 23, 32] have explored various approaches for handling latent observations, there is a lack of systematic evaluation of their model sensitivity. More importantly, there are no comprehensive studies assessing the accuracy of effect estimates derived from different methods. Prioritizing the model interpretability and adaptability, we selected the linear, Tobit, Cox, and logistic models to conduct a straightforward yet the most comprehensive simulation heretofore that identified the optimal models, with regard to both sensitivity and accuracy of effect estimates in the context of GWAS.

By assessing the models’ robustness in terms of detection power, estimation accuracy, and efficiency against the four varying parameters—sample size, censored proportion, effect size, and MAF—that covered most practical scenarios for GWAS, we found that the linear and Tobit models showed comparably high sensitivity and concordance with each other. Specifically, already with a 10% censored proportion, the Tobit model performed better in capturing accurate effect estimates, while the linear model would underestimate the effect size, of which the relative error could be as large as 67% when the censored proportion reached 90%. However, the linear model was by far the most time-efficient method. We therefore suggest a two-step “Linear-Tobit” scheme to achieve optimal sensitivity, accuracy, and efficiency: 1) perform GWAS with any tools that support linear regression with the latent measurements imputed as the average of the corresponding rank-transformed values, for initial discovery of significant variants; 2) reanalyze detected variants using the Tobit regression to obtain unbiased effect estimates for downstream post-GWAS analyses.

Importance of the second step should be emphasized, since the downstream analyses such as MR, PRS, meta-analyses, or multi-cohort comparisons highly rely on accurate effect estimates. For example, we showed hyperbolic increase in the attenuated inflation of MR Wald ratio estimator in relation to underestimated genetic variant-exposure association, which also exhibited superlinear growth with respect to the increasing censored proportion. More specifically, with 50% censored proportions, which applies to >20% of the proteomics measurements in UKB-PPP project [4], the MR estimate is inflated a factor of ∼1.3. Likewise, the PRS, weighted by GWAS estimates, will also be underestimated with severe consequences for its predictive values. In meta-analyses of GWAS or comparisons between GWAS studies, variability in the effect estimates due to different censored proportions could easily be interpreted as heterogeneity between populations.

As mentioned earlier, the four models investigated here were chosen for their simple implementation and interpretation. The logistic model, while straightforward in its implementation by treating omics measurements simply as binary phenotypes (above- or below-LOD), showed lower detection power compared to the others as a result of loss in data resolution and high sensitivity to class imbalance. Regarding the Cox model, although its accommodation for censored data [23] was initially considered innovative with the expectation of robust detection quality, besides the poorer performance, it also lacks interpretability, making its effect estimates harder to translate effectively in the context of GWAS. Like other non-Gaussian models investigated in previous studies [16, 32], the Cox model may also be prone to model misspecification, further discouraging its usage in GWAS against censored omics measurements.

To examine the Linear-Tobit scheme as well as the findings from the simulation study in general, we applied the Linear-Tobit scheme to real-world data, along with comparisons to the logistic and Cox models. We selected two biomarkers from the UKB—testosterone and estradiol—with varying censored proportions across population strata (pre- and post-menopausal females). The genetic variants identified were indeed found to have biological association with the corresponding biomarkers (Supplementary Table S2). With a small proportion, such as 7.94%, of censored data, the linear model clearly outperformed the logistic; however, as the censoring rate increased, the detection power of the three models became more similar, and the logistic model even identified genetic variants that were likely deviating from the additive genetic assumption (Supplementary Table S3). Nevertheless, in agreement with the simulations, we observed a remarkable increase in the magnitude of effect estimates by the Tobit model compared to those by the linear model when the censored proportion increased, validating its role in refining estimates. Furthermore, though our case studies were not entirely comparable with the other GWASs involving the corresponding hormones due to differences in population stratification and covariates [9, 20], the larger number of significant variants, especially in highly censored data, as well as the reduced bias in the linear effect estimates by Tobit regression, could potentially improve the robustness in post-GWAS analyses, e.g. increasing the number of instruments and resolution for MR, of which most frameworks assume linearity in the exposure-outcome relationship [40]. Additional simulations with multiple SNPs extracted from the case studies showed that the presence of LD did not introduce additional bias that could lead to distorted effect estimates or misleading associations; thus, our conclusions from the single-SNP simulations remained valid.

While the simulation together with the application of Linear-Tobit scheme to real biobank data provided valuable insights, certain limitations must be acknowledged. First, the simulated traits were assumed to be measured with a uniform LOD, overlooking the potential batch effects that could result in randomness of LODs. However, rather than a single pipeline to involve all measurements at once, the Linear-Tobit scheme gives accurate effect estimates to be meta-analyzed across batches or plates and still accounts for variability in detection thresholds. Second, placing Tobit regression as the refinement step represents a practical compromise to enhance the general computational efficiency, which is yet to be improved and implemented into GWAS software. It is important to note that current implementations of the Tobit model do not account for genetic relatedness, which has been incorporated as mixed effects [18, 41–43] in various models. Even though tools with mixed-effect Tobit models are available [44, 45], they do not have feasible integration with the GRMs.

In conclusion, through comprehensive parameter setups designed for biobank-scale GWAS, this study demonstrates that the Linear-Tobit scheme would offer an optimal balance of efficiency, accuracy, and simplicity when analyzing censored biomarker measurements. Case studies with real-world data supported the findings from simulations, particularly highlighting the benefits of combining the linear and Tobit models for analyzing biomarkers with latent measurements. The linear model is widely supported by most GWAS software, including PLINK2 [17], SAIGE [18], and REGENIE [19], and the Tobit model, despite needing more functionality enhancements, is also supported by many statistical tools such as the R packages censReg [31] and VGAM [46]. To further facilitate the practical usage of the Linear-Tobit scheme, we also made publicly available an R package censGWAS that flexibly performs linear-model GWAS and Tobit-model refinement for the clumped or pre-selected variants. It should still be noted that despite the additive genetic model that assumes linear relations between the genotype dosage and phenotype being widely used, it might not always hold, because the traits inherently follow other genetic models with certain variants. Severe censoring, such as over 90%, obscures the full distribution; thus, under such circumstances, using the logistic model could be helpful to provide cross-validation or additional discovery, as also used in previous studies and supported by the GWAS software listed above [9, 20]. Lastly, regarding the complexity of incorporating the GRM into the Tobit model while running it for all variants, we note that the latest GWAS tools have improved the relatedness and efficiency issues by incorporating them as mixed effects [18] or penalizing correlations using ridge regression [19]; thus, we foresee future efforts to improve the implementation of Tobit model into GWAS with similar techniques.

## Supplementary Material

lqaf201_Supplemental_File

## Data Availability

The R source code for censGWAS is available from https://github.com/YADengUU/censGWAS and https://doi.org/10.5281/zenodo.17640993. The dataset supporting the case studies is not deposited in a public repository due to sensitive human data collected by the UK Biobank (https://www.ukbiobank.ac.uk/).
