# Supplementary material for: Improving accuracy in genome-wide association studies: a two-step approach for handling below limit of detection biomarker measurements
Source: NAR Genom Bioinform. 2025 Dec 31;7(4):lqaf201. doi: 10.1093/nargab/lqaf201 (PMC12754788; doi:10.1093/nargab/lqaf201)
Supplement: lqaf201_Supplemental_File [file lqaf201_supplemental_file.pdf]

Supplementary Information for

Improving accuracy in genome-wide association studies: a two-step approach for handling below limit of detection biomarker measurements

Table of Contents

Supplementary Fig. 1...2

Preliminary simulations on GWAS with censored phenotypes

Supplementary Fig. 2...3

Choice of ties.method for linear model

Supplementary Fig. 3...4

The FPR of simulations

Supplementary Fig. 4...5

Simulations regarding the varying effect size and MAF

Supplementary Fig. 5...6

Simulations regarding the varying sample size and MAF

Supplementary Fig. 6...7

Potential inflation in MR Wald ratio estimator arisen from biased variant-exposure estimate

Supplementary Fig. 7...7

Risky usage of the Cox model for GWAS on censored biomarkers

Supplementary Fig. 8...8

Additional benchmarking for the runtime to evaluate model efficiency

Supplementary Fig. 9...9

The pipeline of categorizing premenopausal and postmenopausal participants from the UKB

Supplementary Fig. 10...10

Additional results of the GWAS case studies with UKB data

Supplementary Fig. 11...11

Effects of phenotype censoring on lead and LD-correlated SNPs

Supplementary Table 1...12

Descriptive characteristics of the participants included in the UKB for case studies

Supplementary Table 2...13

Results of the GWAS case studies with the UKB data

Supplementary Table 3...16

Descriptive statistics of biomarker levels by GWAS-identified variants.

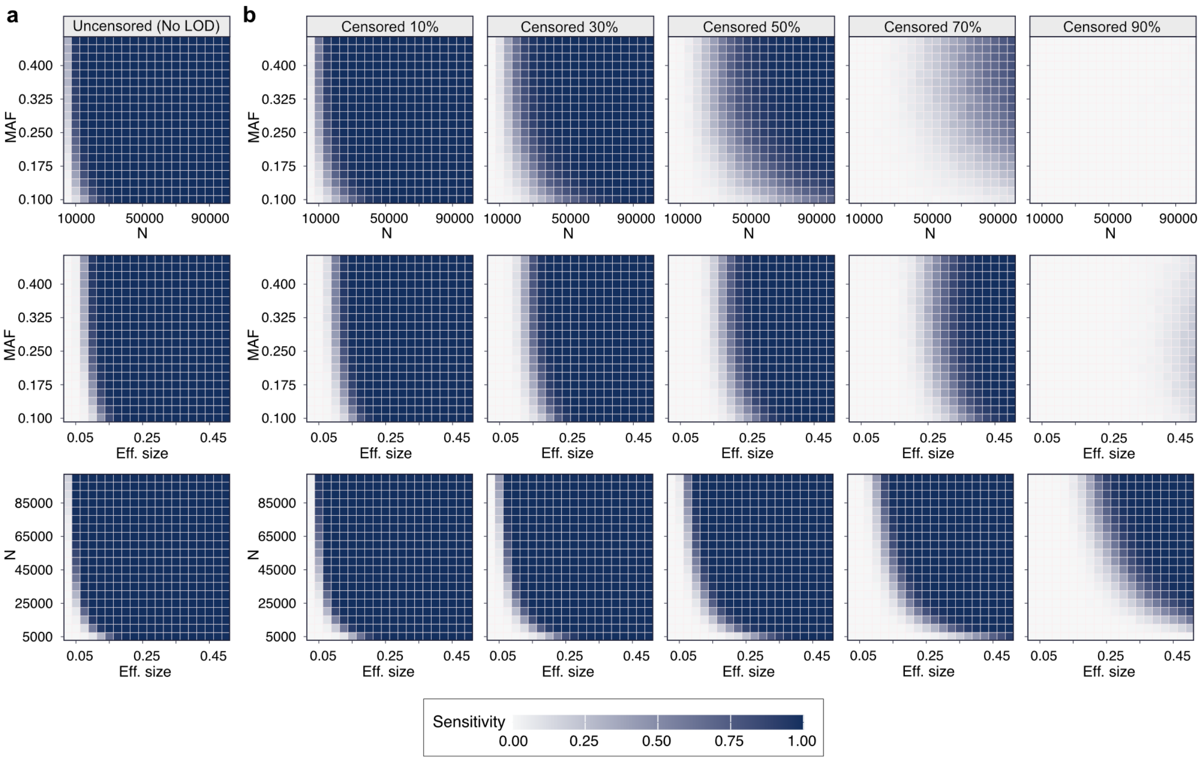

Supplementary Fig. 1 | Preliminary simulations on GWAS with censored phenotypes. In addition to the proportion of censored phenotype measurements, we incorporated three crucial factors relevant to the GWAS outcomes – the sample size (N), the effect size (Eff. size) and minor allele frequency (MAF) of the tested variant, as shown in each row we used a series of values to see how they impacted the detection power. Before adding the artificial LOD, a baseline simulation without any censoring was conducted **(a)** to serve as a reference point, showing the constraints in technical aspects, not due to censoring. We also tested the performance of using only the observed measurements above LOD and showed that truncation should not be a preferred approach **(b)**. The linear model was used at this stage.

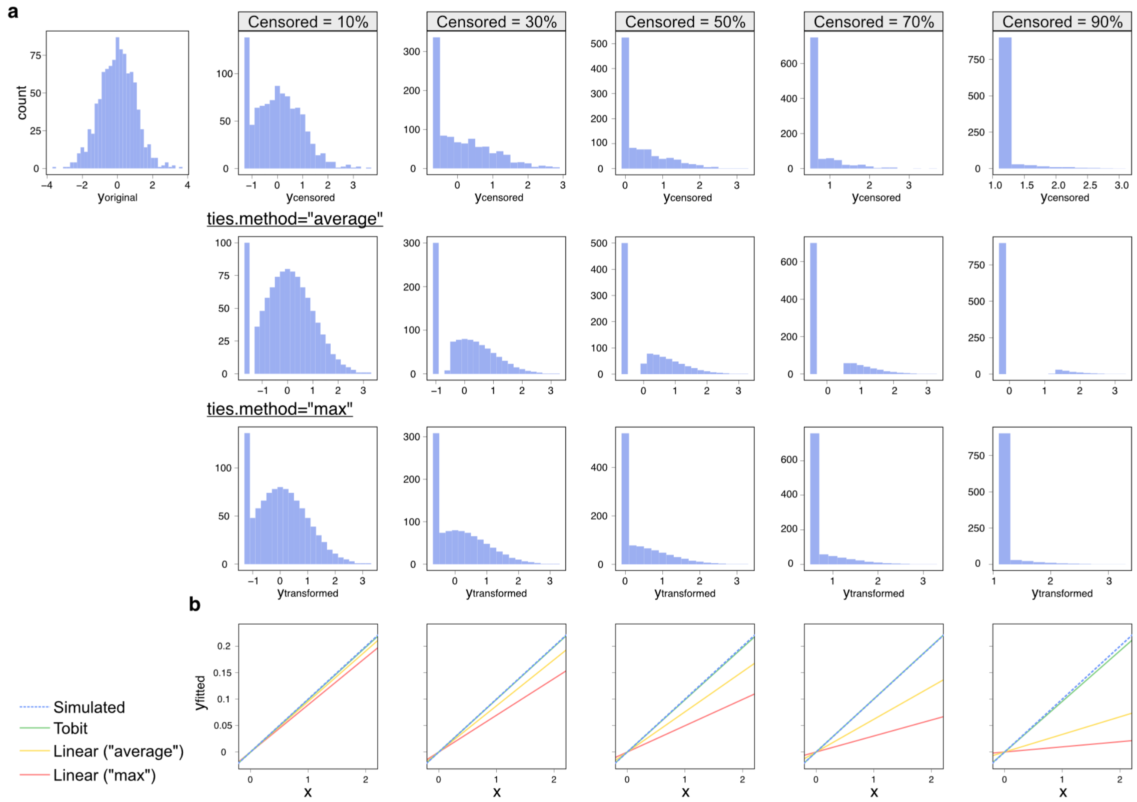

Supplementary Fig.2 | Choice of ties.method for linear model. We ran 500 simulations to generate data of sample size of 1000 with $x \sim \mathcal{N}(0,1)$, $\beta = 0.1$, $y = \beta \cdot x + \mathcal{N}(0,1)$ to test the different ties.method for running inverse normal rank-based transformationon the dependent variable for the linear model. **(a)** Histograms of one of the simulated sets of the dependent variables, with the second and third rows showing the distribution with ties.method="average" and "max", respectively. With "average", the least value becomes the transformed mean below LOD; with "max", the least value is the transformed LOD value. **(b)** Comparison of the slopes that were simulated (blue, dotted), estimated by Tobit (green), by linear model with the least value being the transformed mean below LOD, i.e., ties.method="average" (yellow) and as the transformed LOD value, i.e., ties.method="max" (red).

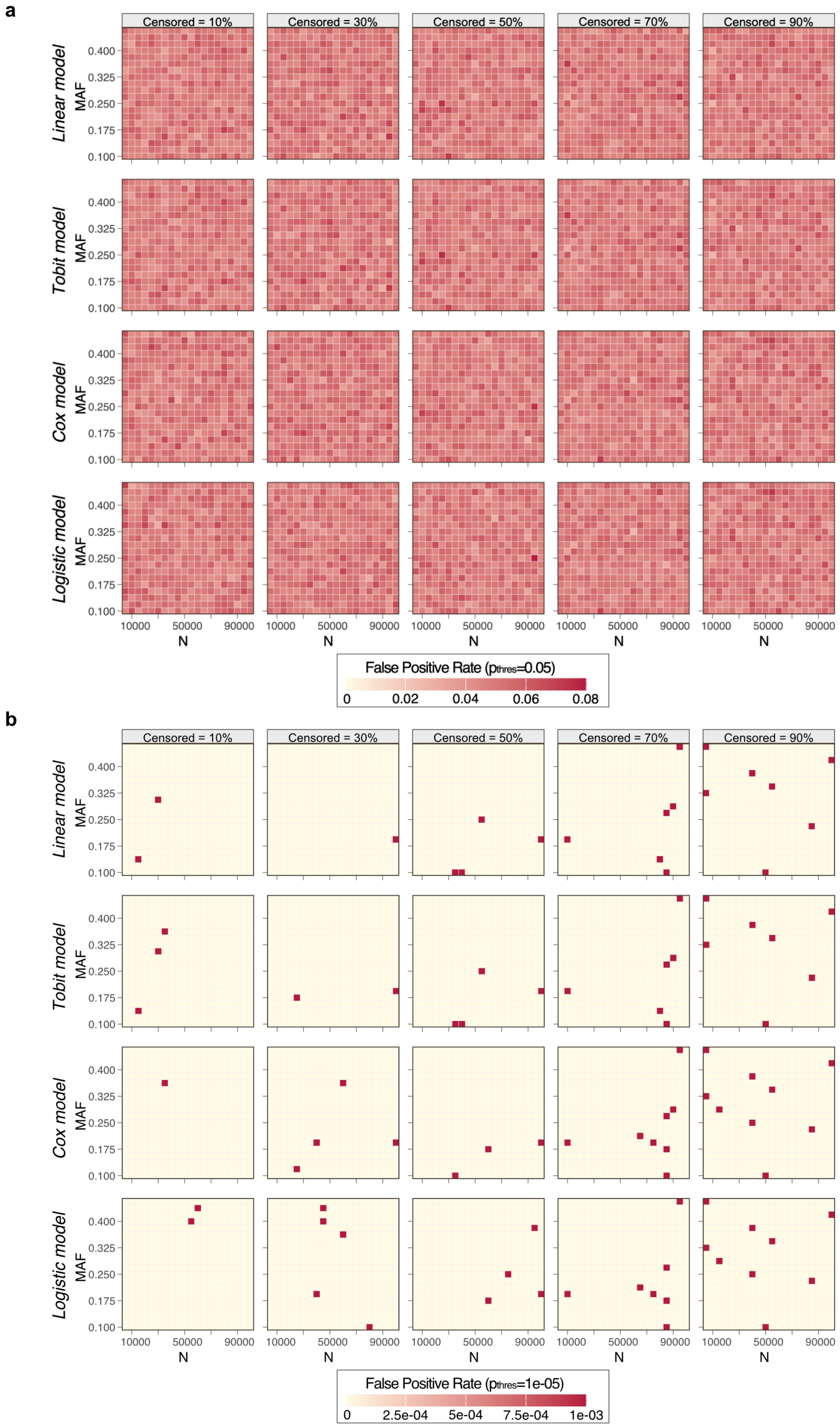

Supplementary Fig. 3 | The FPR of simulations. Using trials with effect size fixed to 0, the first cutoff p=0.05 **(a)** was used for initial validation of the simulation setups, as expecting to observe false positive rate (FPR) having the 95% CIs to be (3.65%, 6.35%); the second cutoff p=1e-05 **(b)** is the commonly used threshold for suggestive association in GWAS, calculated to investigate the impact of censoring and modeling approach on FPR. The most common GWAS significant cutoff 5e-08 was not used because in our simple setup it would result in FPR=0 for all scenarios and thus failed to provide any insights.

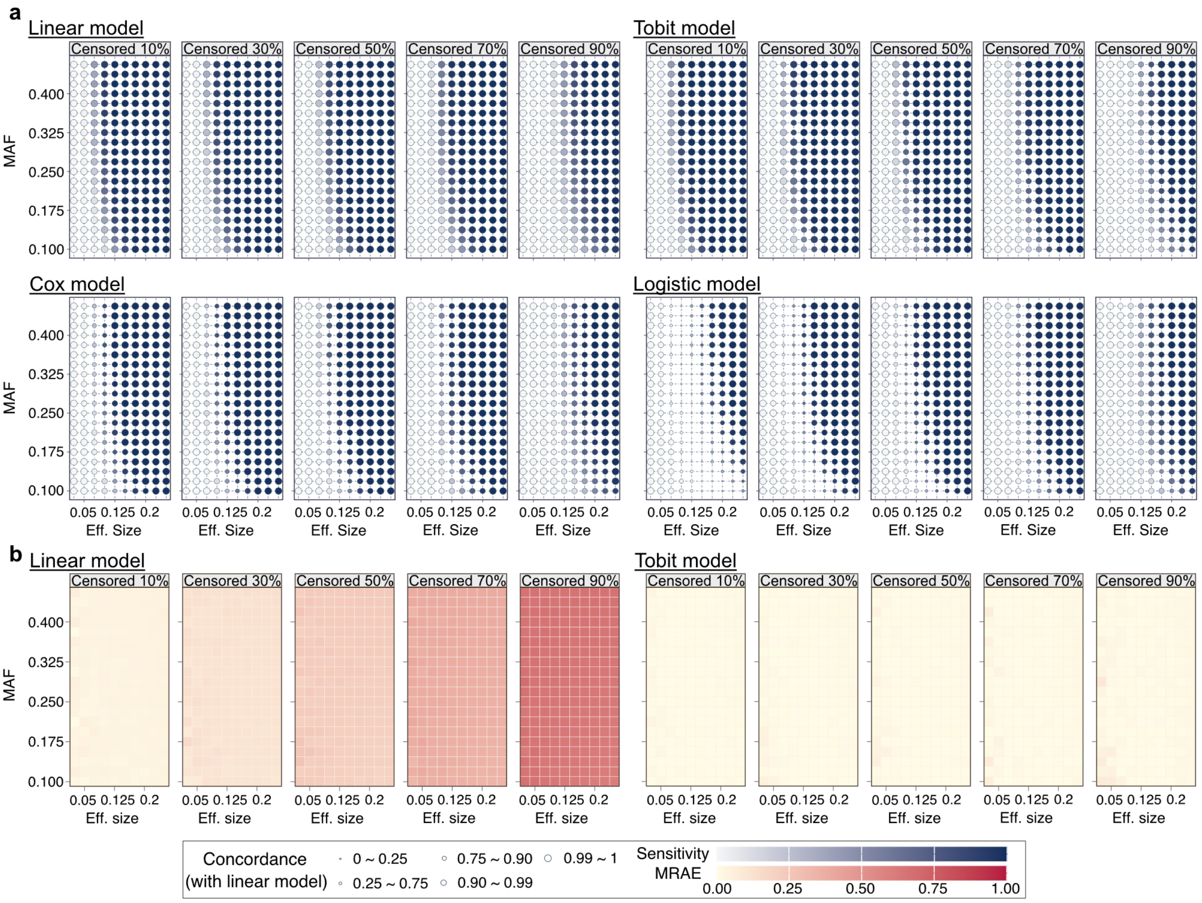

Supplementary Fig. 4 | Simulations regarding the varying effect size and MAF. With the sample size fixed at N=10,000, we sought into how the properties of the tested variant could impact the detection quality along with the changing censored proportion. We compared the sensitivity of the four models, and for the Tobit, Cox, and logistic models, we checked their concordance with the linear model **(a)**. As the linear and Tobit models are designed to estimate linear association, we calculated the MRAE in the estimated effect **(b)**.

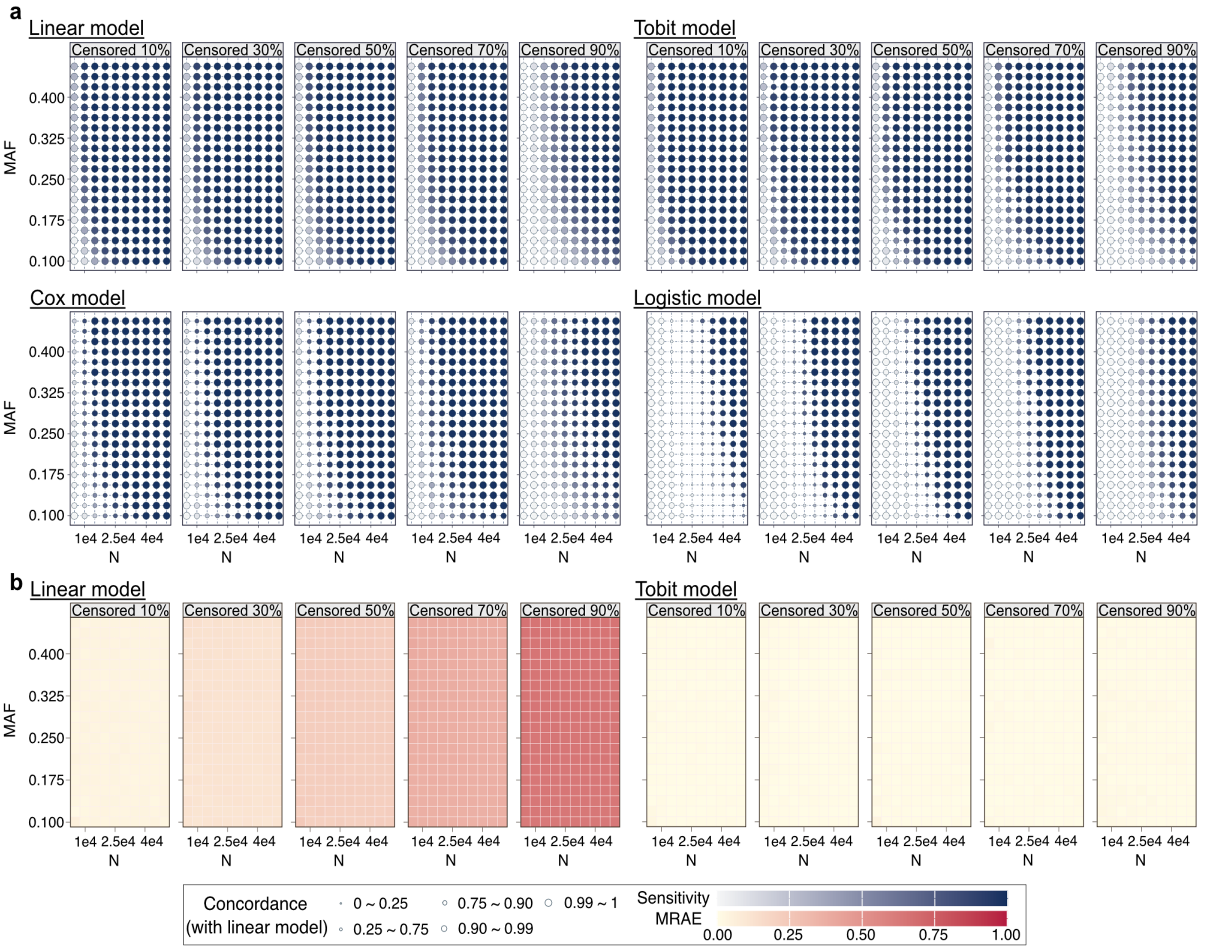

Supplementary Fig. 5 | Simulations regarding the varying sample size and MAF. With the effect size of the SNP fixed at 0.1, we sought into how its minor allele frequency (MAF) and the sample size (N) could impact the detection quality along with the changing censored proportion, with the sensitivity and concordance (with the linear model) of the four models calculated **(a)**, as well as the MRAE in the estimated effects by the linear and Tobit models **(b)**.

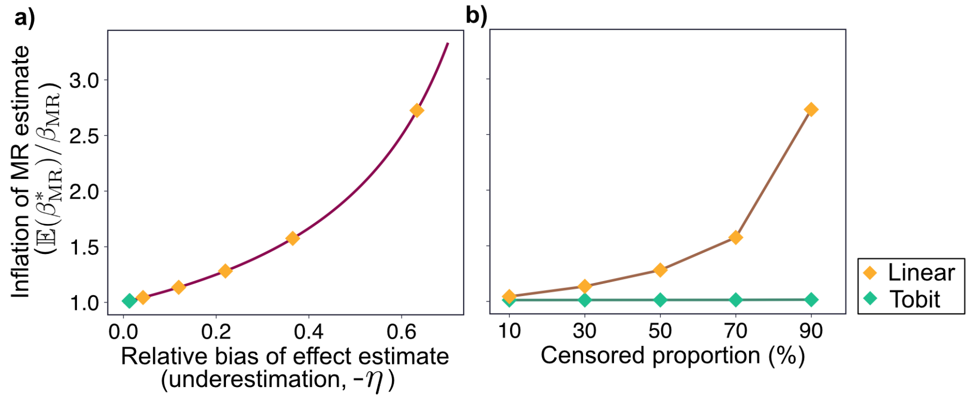

Supplementary Fig. 6 | Potential inflation in MR Wald ratio estimator arisen from biased variant-exposure estimate. For the inflation factor in the causal estimator $\mathbb{E}(\beta^*_{MR})/\beta_{MR}$ we have deduced that, as shown in the Methods, it has a hyperbolic growth in relation to the relative bias η in the estimated variant-exposure association. In **(a)**, the purple curve plots relation $\mathbb{E}(\beta^*_{MR})/\beta_{MR} = (1+\eta)^{-1}$ against -η, as the systematic underestimation ($\eta < 0$) was observed; the markers represent the theoretical inflation resulted from the corresponding relative bias across simulated scenarios for a specific censored proportion (i.e., 10~90%). For further clarity, in **(b)**, we directly plot the MR inflation factor vs the censored proportions, to highlight the accelerating increase of MR estimator bias caused by censoring if not appropriately handled, such as by Tobit regression. Markers in yellow denote the data points for the linear model and in green the Tobit model.

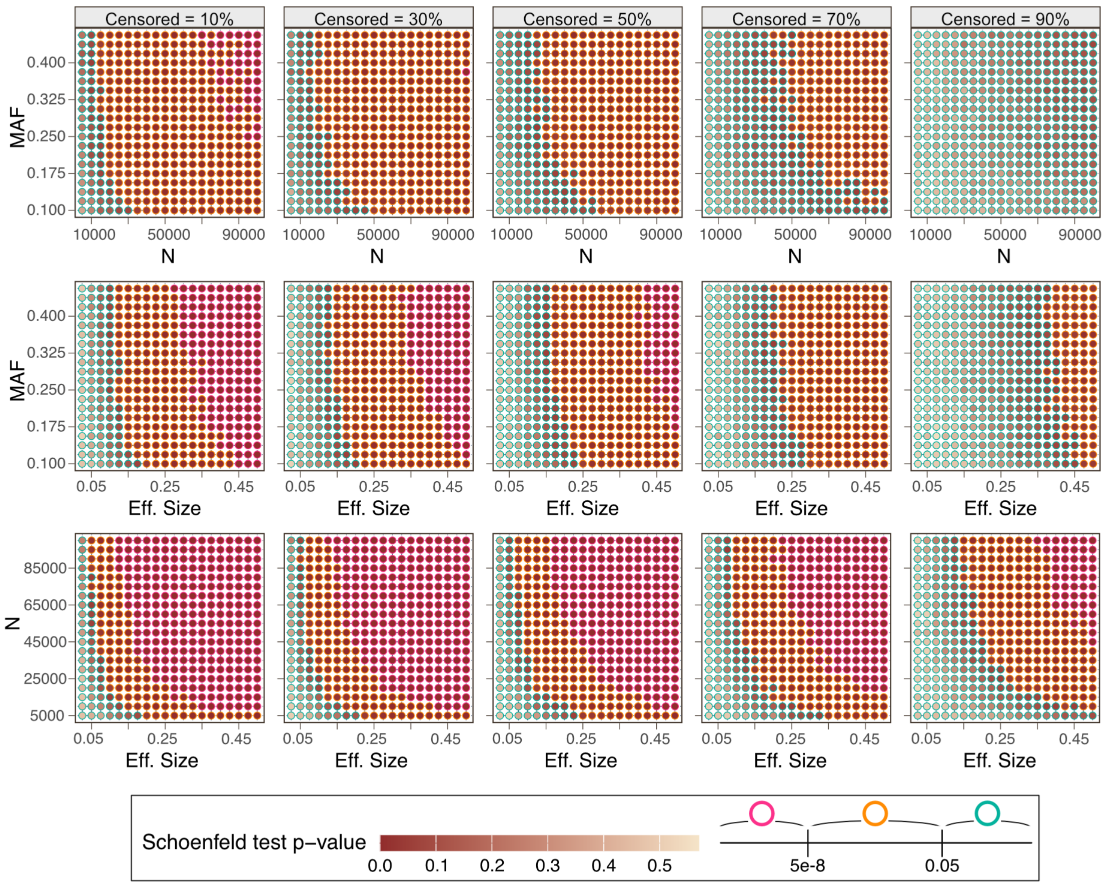

Supplementary Fig. 7 | Risky usage of the Cox model to accommodate censored phenotypes. Running the Schoenfeld's test for using the Cox model showed the risk of violation of the PH assumption in many scenarios. Even if the model was able to yield acceptable outcomes, its usage should be taken with caution and even not be recommended.

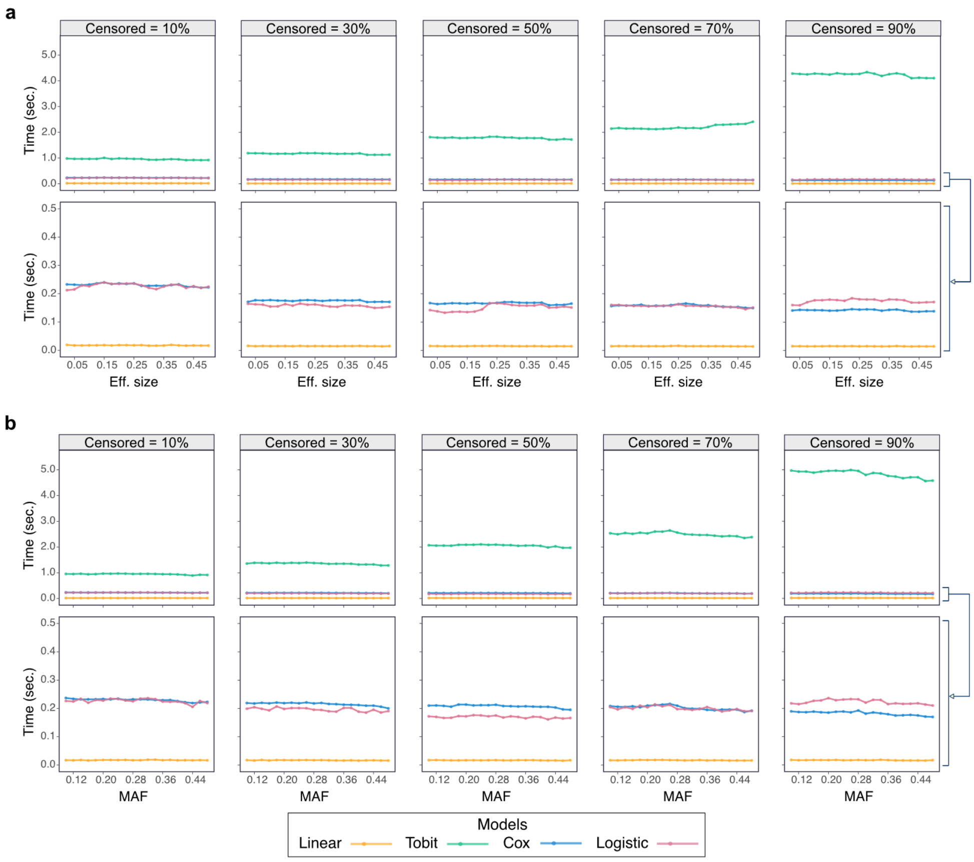

Supplementary Fig. 8 | Additional benchmarking for the runtime to evaluate model efficiency. Unlike the sample size, neither the effect size (Eff. size) **(a)** nor the minor allele frequency (MAF) **(b)** of the tested genetic variant influenced the model efficiency. However, we could again observe that the Tobit model had an increasing computational demand as there were more measurements being censored.

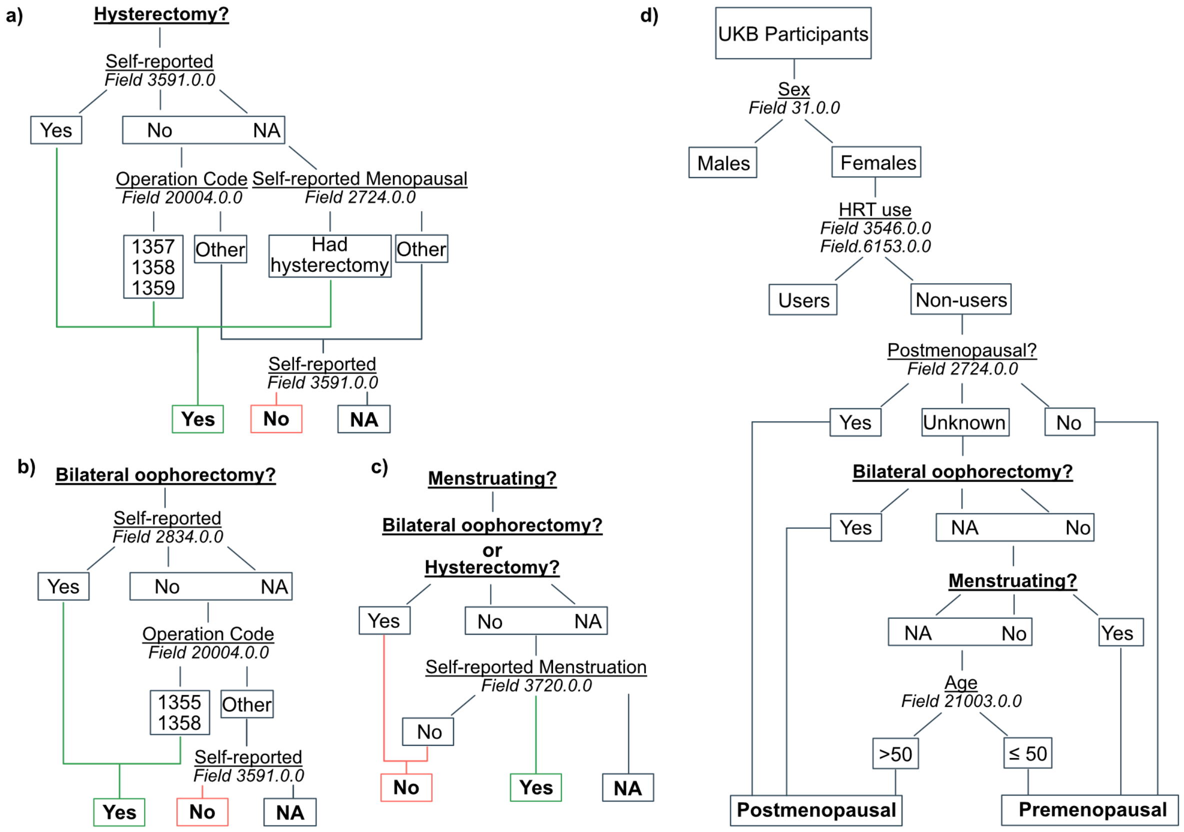

Supplementary Fig. 9 | The pipeline for categorizing participants and related covariates from the UKB. In addition to the self-reported field about the menopausal status, we took the surgical history, menstrual status, and the age into consideration, in order to obtain a larger sample size with more accurate categorization for hysterectomy status **(a)**, bilateral oophorectomy status **(b)**, and menstruation status **(c)**, and based on these we classify the female participants into post- and pre-menopausal groups **(d)**.

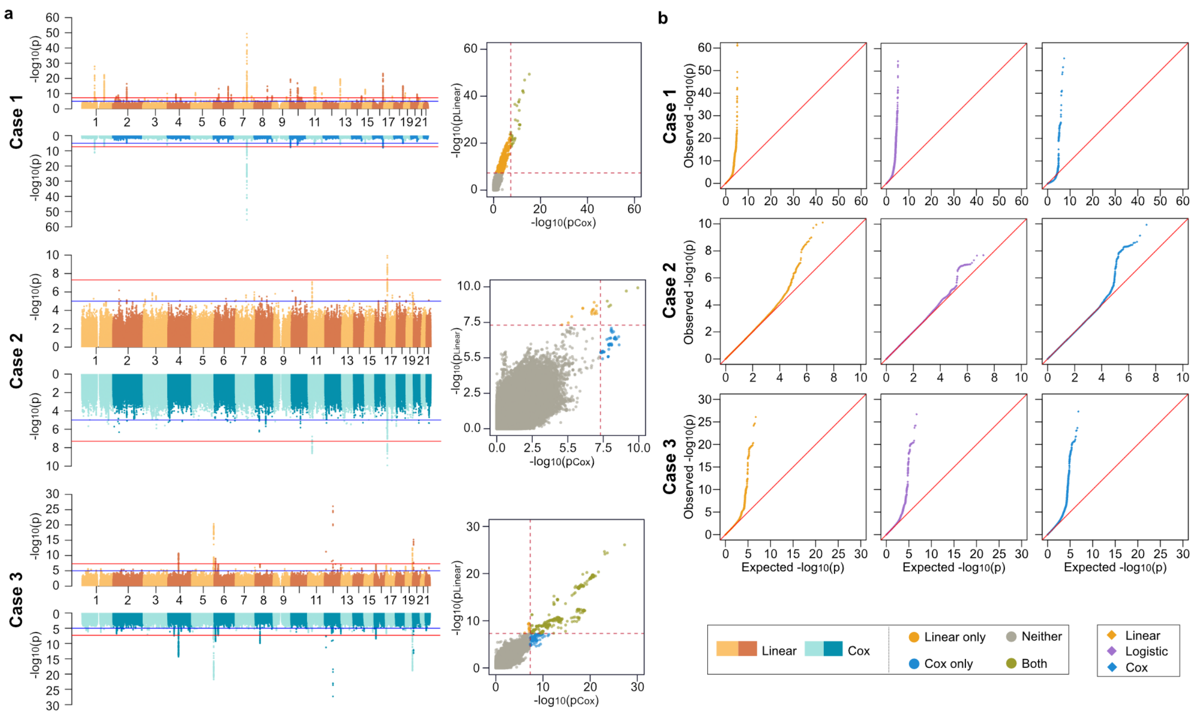

Supplementary Fig. 10 | Additional results of the GWAS case studies with UKB data. We checked the performance of the Cox approach on the three cases **(a)**, even though it was not a recommended strategy, just for validating the simulation conclusions. Like for the comparison of the Linear-Tobit scheme with the logistic model shown in Fig. 3, we also demonstrate the results with Miami plots and the p-value scatter plots for checking model concordance. We assessed the overall performance of all example GWAS for step one of Linear-Tobit scheme, i.e., the linear model, and logistic as well as the Cox model, with the Q-Q plots **(b).**

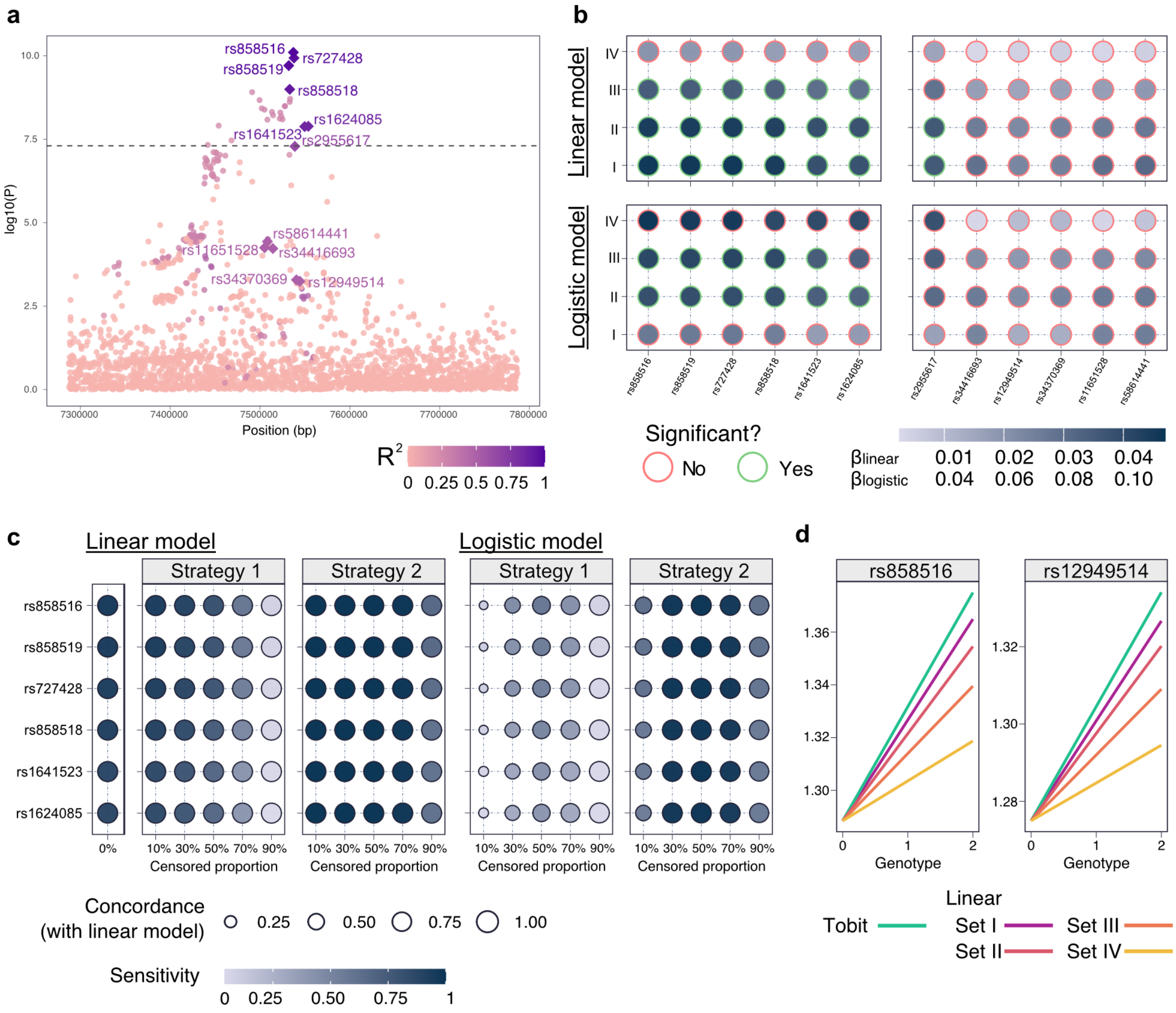

Supplementary Fig. 11 | Effects of phenotype censoring on lead and LD-correlated SNPs. (a) The regional plot of ±250 kb of the lead SNP (rs858516) from Case 2. The shadings represent the magnitude of squared correlations. The ones in high (≥0.8) and medium (0.5~0.8) LD are highlighted. **(b)** By raising the LOD which was initially 175 pmol/L to 300, 500, and 900, we had 4 sets, corresponding to censored proportion of 28.43%, 47.8%, 70.8% and 89.1%. The shading of the bubbles represent the magnitudes of the corresponding regression estimates, and their outlines indicate whether they reached significance ($p<5e-8$) or not, with magenta outline for "No" (insignificant), and green for "Yes" (significant). **(c)** Phenotypes were simulated based on two strategies on the assumed effect sizes, with Strategy 1 having only the lead SNP with non-zero (0.1) effect and Strategy 2 having LD-proportional effects. The shadings represent the sensitivity based on 1000 simulations with sample size of 10000, and the size of the bubbles represent the concordance with the linear model. The sensitivity and concordance for the Tobit model are not shown as they are highly identical to those of the linear model. **(d)** Comparison between the Tobit estimate and linear estimate across different censored proportions modified from Case 2, where rs858516 is the lead SNP at the loci, and rs12949514 is the SNP in the lowest LD with the lead from the medium group.

Supplementary Table 1. Descriptive characteristics of participants of the UKB included for case studies.

	**Testosterone**		**Estradiol**			
	**Premenopausal** (N=42532)		**Premenopausal** (N=40459)		**Postmenopausal** (N=131310)	
	Below LOD	Above LOD	Below LOD	Above LOD	Below LOD	Above LOD
Participants, N (%)	3378 (7.94)	39154 (92.06)	11503 (28.43)	28956 (71.57)	123423 (93.99)	7887 (6.01)
Measured level [a], mean (Q1-Q3)	NA	1.229 (0.831-1.505)	NA	577.3 (289.5-683)	NA	483.6 (223.2-565.6)
Age, mean (Q1-Q3)	45.61 (43-48)	45.26 (43-48)	46.05 (43-49)	45.2 (43-48)	60.51 (51-65)	54.12 (52-56)
BMI, mean (Q1-Q3)	25.45 (22.07-27.58)	26.49 (22.79-29.00)	26.62 (22.88-29.20)	26.29 (22.66-28.09)	27.22 (23.71-29.81)	27.83 (23.64-30.84)
Smoking status, N (%)						
Current	179 (0.42)	3180 (7.48)	988 (2.44)	2138 (5.28)	7370 (5.61)	452 (0.34)
Occasional	75 (0.18)	1121 (2.64)	315 (0.78)	818 (2.02)	1949 (1.48)	133 (0.10)
Previous	1453 (3.42)	16645 (39.14)	4859 (12.01)	12423 (30.71)	60381 (45.98)	3589 (2.73)
Never	1671 (3.93)	18208 (42.81)	5341 (13.20)	13577 (33.56)	53723 (40.91)	3713 (2.83)
Hysterectomy, N (%)						
No	3154 (7.42)	37057 (87.13)	10894 (26.93)	27813 (68.74)	98053 (74.67)	6787 (5.17)
Yes	224 (0.53)	2097 (4.93)	609 (1.51)	1143 (2.83)	25370 (19.32)	1100 (0.84)
Bilateral oophorectomy, N (%)						
No	NA	NA	NA	NA	111748 (85.10)	7453 (5.68)
Yes	NA	NA	NA	NA	11675 (8.89)	434 (0.33)
Is menstruating, N (%)						
No	2759 (6.49)	33325 (78.35)	7978 (19.72)	25400 (62.78)	NA	NA
Yes	619 (1.46)	5829 (13.70)	3525 (8.71)	3556 (8.79)	NA	NA
Oral contraceptive intake, N (%)						
No	2554 (6.00)	35832 (84.25)	9249 (22.86)	27309 (67.50)	NA	NA
Yes	824 (1.94)	3322 (7.81)	2254 (5.57)	1647 (4.07)	NA	NA

[a] Unit of measurement and limit of detection for testosterone (nmol/L; 0.35), for estradiol (pmol/L; 175)

Supplementary Table 2. Result of the GWAS case studies with the UKB data. The SNPs are listed based on the post-clumping hits of GWAS using the linear model. Insignificance is indicated by text in grey.

Case I. *Testosterone in premenopausal females.*

				Models – Estimate (95% CIs) / P-value			
Chr. Pos	SNP ID	Gene	Eff Allele (Freq.)	Linear	Tobit	Cox (Time-to-event)	Logistic
1: 101738121	rs78900934	DHEAS	A (0.306)	0.074 (0.061, 0.087)<br>1.282E-28	0.075 (0.061, 0.088)<br>1.435E-28	0.048 (0.034, 0.061)<br>1.212E-11	0.204 (0.147, 0.261)<br>3.047E-12
1: 179310439	rs7547733	SOAT1	T (0.202)	-0.045 (-0.06, -0.03)<br>2.751E-09	-0.046 (-0.061, -0.031)<br>2.751E-09	-0.024 (-0.039, -0.008)<br>3.884E-03	-0.108 (-0.169, -0.046)<br>6.191E-04
2: 111934107	rs590097	MIR4435-2HG	T (0.352)	-0.054 (-0.066, -0.041)<br>3.830E-17	-0.055 (-0.067, -0.042)<br>2.887E-17	-0.029 (-0.043, -0.016)<br>1.233E-05	-0.115 (-0.168, -0.063)<br>1.599E-05
2: 43533214	rs6719534	THADA	G (0.427)	0.038 (0.026, 0.050)<br>1.073E-09	0.038 (0.026, 0.050)<br>1.048E-09	0.020 (0.007, 0.033)<br>1.888E-03	0.087 (0.036, 0.139)<br>9.33E-04
2: 27730940	rs1260326	GCKR	T (0.393)	-0.036 (-0.048, -0.024)<br>8.787E-09	-0.036 (-0.049, -0.024)<br>8.754E-09	-0.021 (-0.034, -0.008)<br>1.268E-03	-0.080 (-0.131, -0.028)<br>2.471E-03
3: 195942487	rs2342307	SLC51A/<br>PCYT1A	A (0.384)	0.041 (0.029, 0.053)<br>8.561E-11	0.042 (0.029, 0.054)<br>7.175E-11	0.031 (0.018, 0.044)<br>2.743E-06	0.066 (0.013, 0.119)<br>1.415E-02
4: 69333810	rs2603192	TMPRSS11E	C (0.414)	0.039 (0.027, 0.052)<br>2.682E-10	0.040 (0.028, 0.052)<br>2.666E-10	0.015 (0.002, 0.028)<br>2.012E-02	0.098 (0.045, 0.150)<br>2.526E-04
5: 35183831	rs113722563	PRLR	A (0.012)	-0.187 (-0.242, -0.132)<br>2.822E-11	-0.191 (-0.247, -0.135)<br>2.243E-11	-0.088 (-0.145, -0.031)<br>2.430E-03	-0.385 (-0.586, -0.184)<br>1.717E-04
6: 144298733	rs794605	FAM184A	T (0.243)	-0.056 (-0.070, -0.043)<br>2.075E-15	-0.057 (-0.071, -0.043)<br>1.802E-15	-0.028 (-0.042, -0.013)<br>1.559E-04	-0.068 (-0.127, -0.010)<br>2.165E-02
6: 43280028	rs6458331	ZNF318	C (0.275)	-0.052 (-0.065, -0.038)<br>5.830E-14	-0.052 (-0.066, -0.039)<br>5.159E-14	-0.026 (-0.040, -0.012)<br>2.228E-04	-0.074 (-0.130, -0.018)<br>9.956E-03
6: 144298733	rs9376802	PLAGL1	C (0.379)	-0.039 (-0.051, -0.027)<br>6.036E-10	-0.040 (-0.052, -0.027)<br>5.206E-10	-0.019 (-0.032, -0.006)<br>3.360E-03	-0.080 (-0.132, -0.028)<br>2.597E-03
7: 99332948	rs45446698	CYP3A4/<br>CYP3A7	G (0.041)	-0.461 (-0.491, -0.432)<br>1.879E-201	-0.475 (-0.506, -0.445)<br>1.105E-205	-0.251 (-0.282, -0.220)<br>3.489E-56	-0.968 (-1.063, -0.872)<br>2.363E-08
7: 137790882	rs10274304	AKR1D1	G (0.219)	-0.043 (-0.058, -0.029)<br>5.297E-09	-0.044 (-0.058, -0.029)<br>5.439E-09	-0.026 (-0.041, -0.011)<br>5.403E-04	-0.082 (-0.142, -0.021)<br>8.154E-03
7: 73037956	rs34060476	MLXIPL	G (0.134)	0.049 (0.032, 0.067)<br>2.960E-08	0.050 (0.032, 0.068)<br>2.835E-08	0.026 (0.008, 0.045)<br>5.323E-03	0.154 (0.077, 0.232)<br>9.973E-05
8: 105978368	rs11774829	ZFPM2	A (0.104)	0.080 (0.061, 0.100)<br>1.429E-15	0.081 (0.061, 0.101)<br>1.601E-15	0.044 (0.023, 0.066)<br>6.036E-05	0.199 (0.110, 0.289)<br>1.248E-05
8: 143992864	rs3802230	CYP11B2	C (0.463)	0.036 (0.024, 0.048)<br>4.382E-09	0.036 (0.024, 0.048)<br>4.374E-09	0.021 (0.009, 0.034)<br>9.781E-04	0.082 (0.031, 0.133)<br>1.524E-05
9: 114822868	rs4979077	SUSD1	C (0.409)	-0.035 (-0.048, -0.023)<br>1.372E-08	-0.036 (-0.048, -0.024)<br>1.244E-08	-0.017 (-0.030, -0.004)<br>8.702E-03	-0.100 (-0.152, -0.049)<br>1.348E-04
10: 5062752	rs36032941	AKR1C2/	A (0.296)	-0.062 (-0.075, -0.049)	-0.063 (-0.076, -0.049)	-0.040 (-0.054, -0.026)	-0.105 (-0.160, -0.050)

		AKR1C3		3.469E-20	3.483E-20	1.846E-08	1.700E-04
10: 61469538	rs1171614	SLC16A9	T (0.234)	-0.062 (-0.076, -0.048)<br>9.198E-18	-0.063 (-0.077, -0.049)<br>7.575E-18	-0.038 (-0.052, -0.023)<br>6.168E-07	-0.144 (-0.202, -0.086)<br>1.215E-06
10: 64878118	rs6479877	NRBF2/<br>RNU6-543P	G (0.440)	0.047 (0.035, 0.059)<br>2.045E-14	0.048 (0.036, 0.0602)<br>1.712E-14	0.029 (0.016, 0.042)<br>7.336E-06	0.136 (0.084, 0.188)<br>2.580E-07
10: 94460687	rs10882100	HHEX	C (0.481)	-0.033 (-0.045, -0.021)<br>4.389E-08	-0.034 (-0.046, -0.022)<br>4.538E-08	-0.019 (-0.031, -0.006)<br>3.021E-03	-0.105 (-0.155, -0.054)<br>4.876E-05
11: 72317557	rs171021	PDE2A	T (0.298)	-0.052 (-0.065, -0.039)<br>6.322E-15	-0.053 (-0.066, -0.040)<br>4.960E-15	-0.026 (-0.039, -0.012)<br>2.180E-04	-0.077 (-0.132, -0.023)<br>5.617E-03
11: 62842822	rs377204392	SLC22A24/<br>TUBAP7	T (0.065)	0.096 (0.072, 0.120)<br>6.984E-15	0.097 (0.073, 0.122)<br>6.617E-15	0.063 (0.037, 0.090)<br>3.328E-06	0.155 (0.047, 0.264)<br>5.063E-03
11: 123438118	rs850293	GRAMD1B	C (0.112)	0.064 (0.045, 0.083)<br>4.148E-11	0.065 (0.046, 0.084)<br>4.078E-11	0.042 (0.022, 0.063)<br>5.767E-05	0.221 (0.135, 0.308)<br>5.424E-07
13: 22298923	rs606950	FGF9/<br>RN7SL766P	G (0.387)	-0.058 (-0.070, -0.046)<br>2.755E-20	-0.059 (-0.071, -0.046)<br>2.767E-20	-0.031 (-0.044, -0.018)<br>3.346E-06	-0.102 (-0.154, -0.050)<br>1.161E-04
14: 98552503	rs1121703	LINC01550/<br>LINC02295	T (0.399)	0.037 (0.024, 0.049)<br>4.121E-09	0.037 (0.025, 0.050)<br>4.296E-09	0.022 (0.010, 0.035)<br>6.474E-04	0.103 (0.051, 0.156)<br>8.933E-05
15: 40360741	rs7181230	SRP14-DT	G (0.349)	0.047 (0.034, 0.059)<br>1.942E-13	0.047 (0.035, 0.060)<br>1.930E-13	0.030 (0.016, 0.043)<br>1.051E-05	0.139 (0.085, 0.193)<br>4.218E-07
16: 81590541	rs58072681	CMIP	C (0.072)	0.119 (0.096, 0.142)<br>6.408E-24	0.120 (0.097, 0.144)<br>7.097E-24	0.073 (0.048, 0.098)<br>1.249E-08	0.318 (0.208, 0.429)<br>1.786E-08
17: 66879927	rs34931250	ABCA8	T (0.062)	-0.073 (-0.098, -0.048)<br>7.202E-09	-0.074 (-0.099, -0.049)<br>7.520E-09	-0.039 (-0.066, -0.013)<br>4.048E-03	-0.116 (-0.217, -0.015)<br>2.464E-02
18: 71937461	rs4892190	CYB5A	A (0.172)	-0.065 (-0.081, -0.049)<br>9.751E-16	-0.066 (-0.082, -0.049)<br>1.172E-15	-0.037 (-0.054, -0.021)<br>1.213E-05	-0.139 (-0.204, -0.074)<br>2.560E-05
19: 12502457	rs4804669	ZNF799	A (0.217)	0.049 (0.035, 0.064)<br>4.116E-11	0.050 (0.035, 0.065)<br>3.966E-11	0.027 (0.012, 0.043)<br>5.364E-04	0.113 (0.049, 0.176)<br>5.226E-04
19: 10471462	rs8111359	TYK2	T (0.094)	-0.063 (-0.084, -0.043)<br>2.051E-09	-0.064 (-0.085, -0.043)<br>2.184E-09	-0.031 (-0.053, -0.009)<br>5.574E-03	-0.124 (-0.208, -0.039)<br>4.016E-03
20: 48905114	rs6063496	CEBPB	C (0.274)	-0.043 (-0.056, -0.030)<br>2.976E-10	-0.044 (-0.057, -0.030)<br>2.996E-10	-0.027 (-0.041, -0.013)<br>1.686E-04	-0.092 (-0.148, -0.036)<br>1.217E-03

Case II. *Estradiol in premenopausal females*

				Models – Estimate (95% CIs) / P-value			
Chr. Pos	SNP ID	Gene	Eff Allele (Freq.)	Linear	Tobit	Cox (Time-to-event)	Logistic
7: 85307752	rs143125680	N/A	A (0.015)	0.101 (0.052, 0.149)<br>5.014E-05	0.062 (0.022, 0.101)<br>2.357E-03	0.076 (0.013, 0.139)<br>1.755E-02	0.448 (0.291, 0.605)<br>2.049E-08
11: 30226356	rs11031005	FSHB	C (0.145)	-0.043 (-0.059, -0.028)<br>8.165E-08	-0.039 (-0.052, -0.026)<br>4.753E-09	-0.059 (-0.080, -0.039)<br>8.504E-09	-0.076 (-0.123, -0.030)<br>1.275E-03
17: 7537098	rs858516	SHBG	C (0.443)	-0.044 (-0.056, -0.032)	-0.050 (-0.064, -0.036)	-0.044 (-0.058, -0.029)	-0.067 (-0.099, -0.033)

				9.910E-13	1.261E-12	2.139E-09	8.933E-05
19: 55827175	rs34962991	TMEM150B	A (0.355)	-0.027 (-0.039, -0.016) 4.893E-06	-0.019 (-0.029, -0.009) 1.215E-04	-0.028 (-0.043, -0.013) 2.43E-04	-0.096 (-0.130, -0.061) 4.795E-08

Case III. *Estradiol in postmenopausal females*

				Models – Estimate (95% CIs) / P-value			
Chr. Pos	SNP ID	Gene	Eff Allele (Freq.)	Linear	Tobit	Cox (Time-to-event)	Logistic
4: 84423359	rs7671387	N/A	A (0.489)	0.010 (0.007, 0.012) 1.857E-11	0.055 (0.039, 0.071) 4.587E-11	0.106 (0.078, 0.134) 8.104E-14	0.116 (0.082, 0.149) 1.500E-11
5: 176465099	rs2336718	ZNF346	T (0.487)	0.014 (0.011, 0.016) 4.508E-21	0.075 (0.058, 0.091) 4.715E-19	0.141 (0.113, 00.169) 1.695E-22	0.165 (0.131, 0.198) 1.427E-21
6: 10977049	rs9393822	N/A	C (0.264)	-0.010 (-0.013, -0.007) 9.537E-10	-0.057 (-0.075, -0.038) 3.455E-09	-0.101 (-0.132, -0.069) 5.263E-10	-0.121 (-0.159, -0.082) 1.012E-09
6: 31826414	rs2507954	NEU1	G (0.331)	0.008 (0.005, 0.011) 7.442E-08	0.051 (0.033, 0.068) 9.052E-09	0.075 (0.045, 0.105) 9.187E-07	0.104 (0.068, 0.140) 9.361E-09
8: 37886623	rs28617731	EIF4EBP1	C (0.224)	0.009 (0.006, 0.013) 6.610E-08	0.053 (0.034, 0.072) 6.683E-08	0.111 (0.077, 0.145) 1.193E-10	0.121 (0.081, 0.160) 1.964E-09
12: 66704225	rs75770066	HELB	G (0.033)	0.043 (0.035, 0.051) 7.880E-27	0.222 (0.181, 0.263) 6.112E-26	0.484 (0.397, 0.570) 1.620E-31	0.457 (0.374, 0.539) 1.949E-27
12: 10875928	rs77100210	YBX3	C (0.053)	0.022 (0.016, 0.029) 5.634E-12	0.117 (0.082, 0.152) 6.572E-11	0.235 (0.169, 0.302) 5.215E-12	0.242 (0.170, 0.313) 2.763E-11
12: 57146069	rs2277339	PRIM1	G (0.104)	-0.014 (-0.018, -0.009) 8.467E-09	-0.075 (-0.102, -0.047) 1.140E-07	-0.141 (-0.186, -0.096) 6.768E-10	-0.171 (-0.228, -0.113) 5.313E-09
16: 11919959	rs4493057	BCAR4	C (0.341)	0.008 (0.005, 0.011) 1.838E-07	0.049 (0.032, 0.066) 1.580E-08	0.087 (0.058, 0.117) 5.768E-09	0.100 (0.065, 0.135) 2.070E-08
17: 5333380	rs8079643	RPAIN	T (0.221)	0.009 (0.006, 0.012) 1.899E-07	0.055 (0.036, 0.075) 2.392E-08	0.087 (0.054, 0.121) 4.058E-07	0.117 (0.077, 0.156) 7.139E-09
19: 55831088	rs2384688	TMEM150B	G (0.495)	-0.010 (-0.013, -0.008) 3.753E-13	-0.061 (-0.077, -0.044) 3.779E-13	-0.123 (-0.151, -0.096) 4.206E-18	-0.143 (-0.177, -0.110) 5.866E-17
20: 5948227	rs16991615	MCM8	A (0.065)	0.041 (0.036, 0.047) 4.364E-45	0.207 (0.177, 0.238) 2.206E-40	0.453 (0.390, 0.516) 1.030E-50	0.440 (0.379, 0.501) 2.303E-45

Supplementary Table 3. Descriptive statistics of biomarker levels by GWAS-identified variants. The SNPs only identified by the linear model but not by the logistic model are underscored by solid line; those only by the logistic model but not the linear model are highlighted in orange. For each SNP, each row represents the distribution for the major, heterozygous, and minor alleles.

Case I. *Testosterone in premenopausal females.* The overall skewness pre- and post-transformation is 3.703 and 0.004, of which considering only those uncensored (above LOD) is 4.320 and 0.345; the overall kurtosis pre- and post-transformation is 57.510 and 2.665, of which considering only the uncensored is 69.451 and 2.688.

SNP ID	All Data – Original (Rank Transformed)			%Uncensored	Only Uncensored – Original (Rank Transformed)		
	Mean	Skewness	Kurtosis		Mean	Skewness	Kurtosis
	1.133 (0.278)	3.114 (0.027)	44.636 (2.632)	91.202	1.208 (0.441)	3.679 (0.372)	54.967 (2.695)
rs78900934	1.166 (0.338)	3.311 (-0.013)	47.420 (2.701)	92.684	1.231 (0.476)	3.849 (0.327)	56.968 (2.696)
	1.233 (0.434)	4.575 (-0.038)	63.107 (2.734)	93.728	1.292 (0.557)	5.152 (0.294)	72.440 (2.661)
	1.167 (0.337)	2.850 (-0.009)	37.498 (2.658)	92.337	1.235 (0.482)	3.331 (0.328)	45.426 (2.654)
rs7547733	1.149 (0.302)	3.750 (0.024)	52.158 (2.671)	91.640	1.222 (0.458)	4.361 (0.370)	62.586 (2.729)
	1.092 (0.218)	2.024 (0.036)	19.178 (2.490)	90.632	1.168 (0.386)	2.386 (0.338)	23.529 (2.530)
	1.185 (0.365)	4.221 (-0.013)	74.221 (2.686)	92.800	1.250 (0.502)	4.886 (0.323)	88.687 (2.665)
rs590097	1.147 (0.300)	3.807 (0.016)	53.736 (2.673)	91.646	1.220 (0.455)	4.430 (0.361)	64.526 (2.736)
	1.118 (0.260)	1.823 (0.014)	14.713 (2.572)	91.168	1.192 (0.422)	2.149 (0.347)	17.808 (2.600)
	1.141 (0.288)	4.768 (0.036)	78.346 (2.663)	91.498	1.215 (0.446)	5.509 (0.378)	93.177 (2.731)
rs6719534	1.167 (0.335)	3.110 (0.002)	45.467 (2.679)	92.279	1.235 (0.481)	3.637 (0.346)	55.169 (2.689)
	1.178 (0.356)	3.651 (-0.054)	55.727 (2.661)	92.601	1.244 (0.497)	4.271 (0.280)	67.353 (2.638)
	1.179 (0.351)	4.807 (-0.012)	78.982 (2.696)	92.499	1.246 (0.494)	5.526 (0.329)	93.243 (2.703)
rs1260326	1.152 (0.314)	2.654 (0.003)	34.898 (2.654)	91.963	1.223 (0.464)	3.117 (0.344)	42.651 (2.676)
	1.134 (0.278)	3.564 (0.049)	54.635 (2.630)	91.288	1.209 (0.439)	4.169 (0.386)	66.407 (2.693)
	1.136 (0.283)	3.503 (0.034)	47.598 (2.684)	91.477	1.210 (0.441)	4.087 (0.383)	57.431 (2.764)
rs2342307	1.171 (0.340)	4.473 (-0.004)	76.373 (2.675)	92.371	1.239 (0.484)	5.178 (0.337)	91.175 (2.676)
	1.176 (0.357)	1.197 (-0.073)	6.937 (2.589)	92.215	1.246 (0.506)	1.417 (0.264)	8.039 (2.528)
	1.136 (0.292)	2.465 (-0.002)	32.647 (2.613)	91.800	1.206 (0.443)	2.922 (0.333)	40.397 (2.622)
rs2603192	1.164 (0.323)	4.667 (0.017)	74.337 (2.684)	91.867	1.236 (0.476)	5.377 (0.361)	74.337 (2.737)
	1.188 (0.367)	2.661 (-0.002)	28.581 (2.693)	92.867	1.252 (0.503)	2.661 (0.329)	28.581 (2.678)
	1.163 (0.328)	3.757 (0.002)	58.469 (2.668)	92.158	1.232 (0.475)	4.375 (0.341)	70.437 (2.686)
rs113722563	1.042 (0.129)	0.919 (0.045)	4.151 (2.463)	87.124	1.144 (0.356)	1.077 (0.408)	4.671 (2.644)
	0.748 (-0.287)	0.706 (0.706)	1.5 (1.5)	100	0.748 (-0.287)	0.706 (0.706)	1.5 (1.5)
	1.175 (0.346)	3.275 (-0.017)	45.109 (2.664)	92.243	1.244 (0.494)	3.828 (0.326)	54.528 (2.664)
rs794605	1.146 (0.301)	4.035 (0.023)	67.938 (2.676)	91.993	1.215 (0.450)	4.696 (0.362)	81.831 (2.718)
	1.096 (0.213)	6.803 (0.117)	134.678 (2.666)	90.873	1.171 (0.376)	7.742 (0.453)	156.446 (2.782)
	1.177 (0.349)	3.649 (-0.016)	52.669 (2.673)	92.364	1.245 (0.495)	4.241 (0.324)	63.157 (2.676)
rs6458331	1.146 (0.301)	4.295 (0.013)	76.516 (2.671)	91.710	1.218 (0.455)	5.028 (0.360)	92.825 (2.721)
	1.109 (0.240)	2.730 (0.088)	29.794 (2.668)	90.953	1.185 (0.403)	3.185 (0.443)	35.996 (2.759)
rs9376802	1.175 (0.351)	3.942 (-0.041)	65.481 (2.661)	92.573	1.241 (0.492)	4.607 (0.290)	79.121 (2.645)

	1.160 (0.318)	4.139 (0.027)	64.166 (2.684)	91.994	1.230 (0.468)	4.779 (0.371)	76.392 (2.726)
	1.125 (0.270)	1.498 (0.046)	9.254 (2.625)	91.382	1.198 (0.428)	1.739 (0.390)	10.799 (2.664)
	1.182 (0.361)	3.834 (-0.018)	59.856 (2.707)	92.852	1.246 (0.497)	4.436 (0.321)	71.424 (2.691)
rs45446698	0.902 (-0.120)	1.563 (0.338)	8.528 (2.683)	83.027	1.015 (0.143)	1.765 (0.715)	10.079 (3.139)
	0.863 (-0.179)	0.841 (0.373)	2.745 (2.174)	83.333	0.966 (0.067)	0.725 (0.510)	2.591 (2.133)
	1.169 (0.341)	3.671 (-0.012)	60.672 (2.676)	92.381	1.238 (0.485)	4.295 (0.331)	73.573 (2.673)
rs10274304	1.146 (0.296)	3.418 (0.030)	44.388 (2.653)	91.598	1.219 (0.452)	3.969 (0.366)	53.219 (2.712)
	1.127 (0.262)	6.274 (0.077)	108.517 (2.711)	91.563	1.199 (0.416)	7.079 (0.414)	124.716 (2.820)
	1.151 (0.309)	3.521 (0.006)	55.206 (2.648)	91.778	1.223 (0.462)	4.135 (0.347)	67.340 (2.681)
rs34060476	1.184 (0.360)	3.438 (0.004)	42.870 (2.702)	92.835	1.248 (0.496)	3.941 (0.338)	50.427 (2.695)
	1.209 (0.391)	9.461 (-0.005)	174.829 (2.924)	93.582	1.268 (0.514)	10.258 (0.361)	190.618 (2.958)
	1.149 (0.305)	3.371 (0.019)	49.088 (2.663)	91.766	1.221 (0.459)	3.945 (0.364)	59.585 (2.703)
rs11774829	1.199 (0.390)	4.873 (-0.059)	88.069 (2.733)	93.337	1.259 (0.519)	5.600 (0.278)	104.025 (2.689)
	1.229 (0.435)	0.885 (-0.103)	4.293 (2.485)	92.857	1.297 (0.577)	1.021 (0.183)	4.681 (2.372)
	1.143 (0.295)	3.950 (0.027)	68.221 (2.668)	91.627	1.216 (0.450)	4.626 (0.373)	82.938 (2.724)
rs3802230	1.158 (0.318)	4.012 (0.010)	61.291 (2.668)	92.031	1.228 (0.468)	4.653 (0.348)	73.343 (2.701)
	1.184 (0.366)	2.691 (-0.034)	34.456 (2.663)	92.794	1.249 (0.504)	3.149 (0.303)	41.681 (2.617)
	1.175 (0.348)	2.353 (-0.010)	23.375 (2.696)	92.424	1.242 (0.492)	2.742 (0.336)	27.990 (2.701)
rs4979077	1.151 (0.310)	3.053 (0.016)	42.559 (2.635)	92.037	1.220 (0.459)	3.567 (0.348)	51.608 (2.647)
	1.143 (0.289)	4.825 (0.023)	76.339 (2.684)	91.067	1.221 (0.455)	5.619 (0.390)	91.469 (2.775)
	1.182 (0.362)	2.771 (-0.039)	35.880 (2.646)	92.639	1.248 (0.502)	3.243 (0.291)	43.438 (2.616)
rs36032941	1.146 (0.294)	4.873 (0.040)	79.770 (2.689)	91.652	1.218 (0.449)	5.602 (0.384)	94.234 (2.764)
	1.113 (0.245)	5.978 (0.066)	121.790 (2.768)	90.963	1.189 (0.409)	6.965 (0.451)	146.103 (2.919)
	1.180 (0.352)	4.502 (-0.007)	73.513 (2.698)	92.507	1.247 (0.495)	5.183 (0.335)	87.055 (2.706)
rs1171614	1.138 (0.291)	2.577 (0.020)	31.705 (2.633)	91.641	1.210 (0.446)	3.037 (0.361)	38.852 (2.663)
	1.097 (0.217)	2.418 (0.065)	21.331 (2.695)	89.979	1.180 (0.398)	2.852 (0.439)	25.926 (2.884)
	1.134 (0.280)	4.030 (0.017)	62.481 (2.652)	91.260	1.209 (0.441)	4.727 (0.363)	75.867 (2.729)
rs6479877	1.167 (0.336)	3.394 (-0.0002)	50.650 (2.661)	92.493	1.233 (0.478)	3.945 (0.332)	60.920 (2.654)
	1.185 (0.363)	3.110 (-0.004)	36.370 (2.716)	92.770	1.250 (0.501)	3.591 (0.341)	43.117 (2.708)
	1.175 (0.349)	4.645 (-0.013)	82.681 (2.708)	92.664	1.240 (0.488)	5.376 (0.334)	98.626 (2.698)
rs10882100	1.161 (0.325)	3.347 (0.007)	49.062 (2.661)	92.174	1.230 (0.472)	3.900 (0.344)	59.227 (2.677)
	1.138 (0.283)	3.026 (0.023)	38.018 (2.620)	91.075	1.215 (0.449)	3.558 (0.363)	46.394 (2.691)
	1.176 (0.348)	4.056 (-0.017)	64.208 (2.684)	92.294	1.245 (0.494)	4.721 (0.329)	77.150 (2.691)
rs171021	1.151 (0.309)	3.655 (0.013)	57.509 (2.638)	91.934	1.223 (0.460)	4.262 (0.345)	69.502 (2.663)
	1.104 (0.237)	1.799 (0.072)	12.149 (2.711)	91.167	1.177 (0.397)	2.094 (0.436)	14.313 (2.816)
	1.152 (0.309)	3.954 (0.019)	62.977 (2.673)	91.923	1.222 (0.460)	4.601 (0.362)	75.793 (2.713)
rs377204392	1.209 (0.407)	2.163 (-0.102)	22.098 (2.632)	92.986	1.274 (0.544)	2.163 (0.225)	26.734 (2.550)
	1.232 (0.480)	0.397 (-0.295)	2.882 (2.644)	94.697	1.281 (0.586)	0.397 (0.067)	3.001 (2.183)
	1.151 (0.307)	3.635 (0.010)	54.261 (2.662)	91.756	1.223 (0.461)	4.252 (0.354)	65.730 (2.706)
rs850293	1.196 (0.379)	4.516 (-0.001)	77.657 (2.724)	93.264	1.257 (0.508)	5.149 (0.330)	90.971 (2.696)
	1.199 (0.408)	1.013 (-0.024)	5.038 (2.607)	94.799	1.246 (0.508)	1.149 (0.330)	5.449 (2.439)

	1.187 (0.365)	4.362 (-0.025)	78.095 (2.662)	92.551	1.255 (0.508)	5.067 (0.311)	93.707 (2.644)
rs606950	1.150 (0.309)	3.073 (-0.005)	40.501 (2.667)	91.784	1.222 (0.463)	3.616 (0.344)	49.393 (2.704)
	1.118 (0.255)	3.052 (0.072)	35.909 (2.688)	91.395	1.190 (0.411)	3.542 (0.416)	43.045 (2.782)
	1.143 (0.293)	3.689 (0.013)	52.443 (2.686)	91.439	1.217 (0.452)	4.323 (0.368)	63.568 (2.764)
rs1121703	1.163 (0.329)	3.596 (0.006)	54.908 (2.672)	92.300	1.231 (0.474)	4.183 (0.344)	66.064 (2.683)
	1.183 (0.363)	4.014 (-0.018)	79.797 (2.672)	92.813	1.247 (0.500)	4.686 (0.306)	96.670 (2.570)
	1.139 (0.287)	4.193 (0.026)	73.225 (2.654)	91.416	1.213 (0.446)	4.919 (0.374)	89.136 (2.713)
rs7181230	1.171 (0.341)	3.251 (-0.008)	43.553 (2.680)	92.327	1.240 (0.487)	3.783 (0.332)	52.334 (2.693)
	1.186 (0.369)	3.749 (-0.022)	56.673 (2.653)	93.268	1.246 (0.497)	4.308 (0.291)	67.036 (2.601)
	1.150 (0.306)	3.725 (0.020)	56.361 (2.672)	91.787	1.222 (0.459)	4.342 (0.365)	67.991 (2.720)
rs58072681	1.214 (0.422)	4.106 (-0.093)	79.865 (2.656)	93.933	1.270 (0.541)	4.769 (0.221)	95.732 (2.527)
	1.333 (0.619)	0.763 (-0.220)	4.250 (2.656)	96.875	1.365 (0.685)	0.858 (-0.019)	4.474 (2.446)
	1.166 (0.331)	3.918 (0.003)	61.094 (2.675)	92.162	1.235 (0.479)	4.552 (0.344)	73.310 (2.696)
rs34931250	1.111 (0.255)	1.191 (0.013)	6.545 (2.580)	91.313	1.184 (0.413)	1.389 (0.343)	7.530 (2.607)
	1.070 (0.197)	0.689 (-0.074)	3.703 (2.393)	90.173	1.148 (0.372)	0.821 (0.244)	4.163 (2.335)
	1.173 (0.345)	3.442 (-0.016)	50.700 (2.658)	92.424	1.241 (0.489)	4.011 (0.317)	61.115 (2.653)
rs4892190	1.132 (0.275)	4.362 (0.038)	75.857 (2.667)	91.280	1.207 (0.436)	5.099 (0.388)	91.837 (2.748)
	1.097 (0.216)	3.994 (0.132)	51.188 (2.871)	90.760	1.173 (0.382)	4.632 (0.548)	61.011 (3.082)
	1.148 (0.301)	4.213 (0.015)	65.027 (2.657)	91.659	1.221 (0.457)	4.894 (0.354)	77.916 (2.713)
rs4804669	1.174 (0.347)	3.767 (-0.003)	64.519 (2.714)	92.559	1.240 (0.488)	4.399 (0.352)	78.061 (2.707)
	1.214 (0.410)	1.900 (-0.068)	14.253 (2.621)	93.096	1.278 (0.545)	2.195 (0.242)	16.627 (2.560)
	1.170 (0.338)	4.024 (-0.006)	63.932 (2.676)	92.249	1.239 (0.485)	4.672 (0.335)	76.640 (2.692)
rs8111359	1.129 (0.269)	3.175 (0.048)	41.295 (2.615)	91.079	1.206 (0.434)	3.708 (0.380)	50.040 (2.689)
	1.154 (0.325)	1.432 (0.156)	6.442 (2.987)	93.782	1.208 (0.440)	1.609 (0.538)	6.979 (3.018)
	1.169 (0.342)	3.371 (-0.034)	57.080 (2.649)	92.356	1.236 (0.487)	3.979 (0.304)	70.096 (2.633)
rs6063496	1.155 (0.309)	4.478 (0.038)	67.902 (2.706)	91.890	1.226 (0.460)	5.149 (0.386)	80.226 (2.768)
	1.115 (0.241)	2.231 (0.125)	16.962 (2.612)	90.783	1.192 (0.408)	2.543 (0.447)	19.763 (2.701)

Case II. *Estradiol in premenopausal females.* The overall skewness pre- and post-transformation is 6.567 and -0.071, of which considering only those uncensored (above LOD) is 6.520 and 1.135; the overall kurtosis pre- and post-transformation is 120.548 and 2.250, of which considering only the uncensored is 114.278 and 4.412.

SNP ID	All Data – Original (Rank Transformed)			%Uncensored	Only Uncensored – Original (Rank Transformed)		
	Mean	Skewness	Kurtosis		Mean	Skewness	Kurtosis
	463.398 (0.868)	6.790 (-0.069)	127.901 (2.237)	71.379	579.036 (1.341)	6.752 (1.128)	121.078 (4.392)
rs143125680	479.125 (0.979)	6.074 (-0.239)	66.668 (2.848)	79.416	557.951 (1.313)	5.971 (1.235)	62.112 (5.342)
	175 (-0.312)	NA (NA)	NA (NA)	0	NA (NA)	NA (NA)	NA (NA)
	471.698 (0.883)	6.608 (-0.067)	121.912 (2.272)	71.936	587.446 (1.349)	6.553 (1.128)	115.382 (4.355)
rs11031005	451.405 (0.852)	7.020 (-0.087)	126.933 (2.219)	71.061	563.970 (1.326)	6.989 (1.148)	119.518 (4.596)
	406.010 (0.784)	2.763 (-0.118)	13.683 (2.047)	69.457	507.725 (1.266)	2.517 (1.078)	11.761 (4.160)
rs858516	479.947 (0.899)	5.998 (-0.085)	96.011 (2.279)	72.408	596.150 (1.360)	5.936 (1.106)	91.106 (4.313)

	463.674 (0.874)	5.342 (-0.082)	86.449 (2.242)	71.807	577.014 (1.340)	5.282 (1.116)	83.333 (4.284)
	441.354 (0.821)	9.903 (-0.029)	229.243 (2.228)	69.798	556.608 (1.311)	9.726 (1.224)	206.529 (4.858)
	466.143 (0.886)	6.505 (-0.103)	121.493 (2.292)	72.525	576.441 (1.339)	6.495 (1.146)	116.464 (4.460)
rs34962991	462.750 (0.866)	5.975 (-0.066)	104.964 (2.218)	71.257	578.820 (1.341)	5.928 (1.105)	100.367 (4.267)
	462.683 (0.840)	8.367 (0.010)	154.666 (2.251)	69.588	588.406 (1.343)	8.086 (1.208)	136.961 (4.792)

Case III. *Estradiol in postmenopausal females.* The overall skewness pre- and post-transformation is 14.035 and 4.372, of which considering only those uncensored (above LOD) is 3.973 and 1.372; the overall kurtosis pre- and post-transformation is 351.957 and 22.988, of which considering only the uncensored is 34.185 and 4.921.

SNP ID	All Data – Original (Rank Transformed)			%Uncensored	Only Uncensored – Original (Rank Transformed)		
	Mean	Skewness	Kurtosis		Mean	Skewness	Kurtosis
	191.618 (-0.226)	11.779 (4.545)	197.493 (24.427)	5.726	465.213 (1.180)	2.893 (1.265)	15.329 (4.532)
rs7671387	194.428 (-0.216)	14.769 (4.323)	424.801 (22.478)	6.377	479.657 (1.193)	4.340 (1.376)	43.758 (4.900)
	196.908 (-0.210)	13.625 (4.288)	288.891 (22.396)	6.653	504.313 (1.215)	3.856 (1.422)	26.959 (5.131)
	191.160 (-0.229)	13.064 (4.633)	248.391 (25.534)	5.591	464.018 (1.175)	3.282 (1.401)	19.090 (5.056)
rs2336718	193.999 (-0.220)	15.831 (4.468)	473.967 (24.048)	6.112	485.880 (1.195)	4.539 (1.400)	45.242 (5.030)
	198.385 (-0.201)	13.185 (4.028)	289.535 (19.794)	7.253	497.436 (1.214)	4.022 (1.377)	31.438 (4.985)
	195.688 (-0.212)	13.243 (4.261)	275.865 (21.992)	6.600	488.439 (1.201)	3.748 (1.409)	26.138 (5.110)
rs9393822	193.326 (-0.222)	15.761 (4.507)	508.766 (24.283)	5.956	482.728 (1.195)	4.529 (1.325)	49.893 (4.723)
	191.353 (-0.229)	11.679 (4.654)	190.224 (25.512)	5.504	472.099 (1.190)	2.760 (1.265)	13.884 (4.296)
	192.836 (-0.223)	15.780 (4.490)	494.271 (24.153)	5.955	474.516 (1.186)	4.526 (1.404)	47.864 (5.116)
rs2507954	195.155 (-0.214)	13.164 (4.294)	284.664 (22.225)	6.491	485.493 (1.198)	3.701 (1.343)	27.318 (4.807)
	196.291 (-0.210)	12.096 (4.222)	218.814 (21.486)	6.649	495.224 (1.214)	3.357 (1.344)	20.482 (4.807)
	193.610 (-0.220)	14.844 (4.447)	415.284 (23.746)	6.111	479.523 (1.190)	4.201 (1.383)	39.990 (4.912)
rs28617731	194.788 (-0.215)	13.397 (4.285)	299.842 (22.078)	6.439	482.335 (1.198)	3.827 (1.343)	29.336 (4.974)
	198.981 (-0.201)	9.937 (4.062)	123.840 (20.109)	7.249	505.820 (1.222)	2.551 (1.391)	10.202 (4.489)
	193.612 (-0.220)	14.587 (4.446)	382.840 (23.753)	6.110	479.599 (1.190)	4.092 (1.396)	36.109 (5.021)
rs75770066	204.862 (-0.175)	8.965 (3.546)	112.066 (15.410)	8.707	517.963 (1.254)	2.711 (1.143)	13.193 (4.086)
	185.474 (-0.235)	6.489 (4.236)	46.290 (19.919)	5.517	364.838 (1.071)	0.575 (0.411)	1.820 (1.710)
	193.687 (-0.219)	12.915 (4.423)	256.979 (23.497)	6.158	478.483 (1.190)	3.441 (1.398)	22.358 (4.912)
rs77100210	199.809 (-0.198)	12.197 (4.001)	238.099 (19.418)	7.297	515.000 (1.237)	3.695 (1.180)	26.014 (4.610)
	209.819 (-0.180)	10.178 (3.861)	127.174 (18.499)	7.877	617.057 (1.360)	2.928 (1.631)	11.796 (5.425)
	194.949 (-0.215)	13.853 (4.306)	353.971 (22.296)	6.427	485.422 (1.200)	3.999 (1.338)	35.999 (4.816)
rs2277339	192.075 (-0.227)	14.758 (4.651)	330.896 (26.048)	5.717	473.686 (1.178)	3.854 (1.519)	26.122 (5.395)
	186.157 (-0.245)	16.550 (5.018)	380.813 (29.644)	4.633	415.814 (1.121)	4.088 (1.788)	24.752 (7.429)
	193.175 (-0.222)	13.602 (4.480)	300.202 (24.034)	5.995	478.142 (1.191)	3.669 (1.380)	26.527 (4.964)
rs4493057	194.836 (-0.215)	15.074 (4.321)	433.287 (22.545)	6.441	482.954 (1.193)	4.436 (1.381)	44.052 (4.975)
	196.593 (-0.209)	11.353 (4.185)	182.252 (21.086)	6.700	497.271 (1.220)	3.087 (1.329)	16.519 (4.679)
rs8079643	193.099 (-0.222)	13.447 (4.479)	289.515 (24.031)	5.995	476.918 (1.189)	3.605 (1.389)	25.344 (4.950)
	196.437 (-0.210)	14.858 (4.234)	424.033 (21.688)	6.679	495.978 (1.210)	4.487 (1.333)	45.173 (4.869)

	195.746 (-0.208)	12.774 (4.138)	249.767 (20.909)	6.981	472.182 (1.178)	3.643 (1.501)	24.105 (5.194)
	195.905 (-0.209)	12.093 (4.137)	222.896 (20.658)	6.821	481.457 (1.199)	3.439 (1.336)	21.812 (4.842)
rs2384688	194.281 (-0.217)	15.478 (4.380)	462.837 (23.072)	6.252	483.389 (1.196)	4.545 (1.372)	46.462 (4.985)
	192.631 (-0.226)	12.849 (4.653)	238.725 (25.902)	5.703	484.173 (1.193)	3.178 (1.393)	18.104 (4.787)
	192.946 (-0.222)	15.086 (4.497)	415.829 (24.278)	5.987	474.756 (1.184)	4.215 (1.415)	38.592 (5.095)
rs16991615	203.754 (-0.183)	9.673 (3.702)	134.125 (16.752)	8.225	524.579 (1.256)	2.885 (1.177)	15.189 (4.220)
	209.388 (-0.150)	8.695 (3.109)	107.520 (12.120)	10.294	509.052 (1.259)	3.164 (1.276)	16.464 (5.036)
